# Acid-catalyzed ring-opening reactions of a cyclopropanated 3-aza-2-oxabicyclo[2.2.1]hept-5-ene with alcohols

**DOI:** 10.3762/bjoc.13.281

**Published:** 2017-12-27

**Authors:** Katrina Tait, Alysia Horvath, Nicolas Blanchard, William Tam

**Affiliations:** 1 Guelph-Waterloo Centre for Graduate Work in Chemistry and Biochemistry, Department of Chemistry, University of Guelph, Guelph, Ontario, N1G 2W1, Canada; 2 Laboratoire de Chimie Moléculaire, ECPM-CNRS UMR7509, University of Strasbourg, 25 rue Becquerel, 67087 Strasbourg, France

**Keywords:** acid catalysis, alcohol nucleophiles, cyclopropanation, heterobicyclic compounds, ring-opening reactions

## Abstract

The acid-catalyzed ring-opening reactions of a cyclopropanated 3-aza-2-oxabicylic alkene using alcohol nucleophiles were investigated. Although this acid-catalyzed ring-opening reaction did not cleave the cyclopropane unit as planned, this represent the first examples of ring-openings of cyclopropanated 3-aza-2-oxabicyclo[2.2.1]alkenes that lead to the cleavage of the C–O bond instead of the N–O bond. Different acid catalysts were tested and it was found that pyridinium toluenesulfonate in methanol gave the best yields in the ring-opening reactions. The scope of the reaction was successfully expanded to include primary, secondary, and tertiary alcohol nucleophiles. Through X-ray crystallography, the stereochemistry of the product was determined which confirmed an S_N_2-like mechanism to form the ring-opened product.

## Introduction

**Scheme 1:**
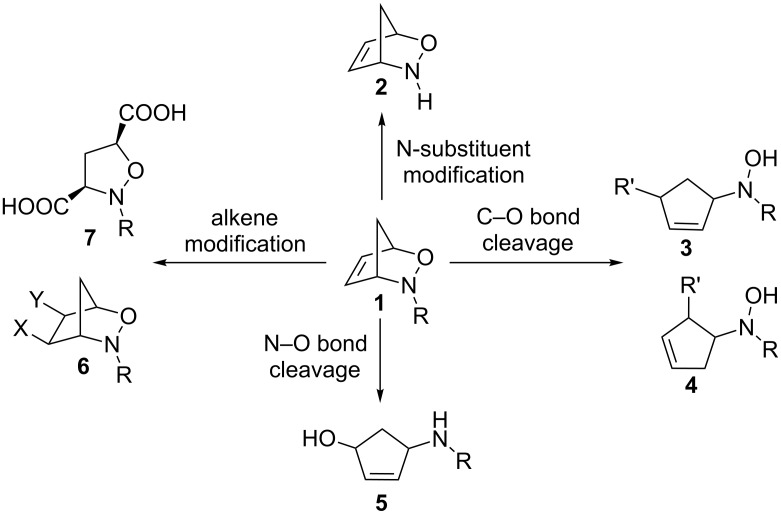
General reaction pathways for 3-aza-2-oxabicyclic alkenes.

**Scheme 2:**
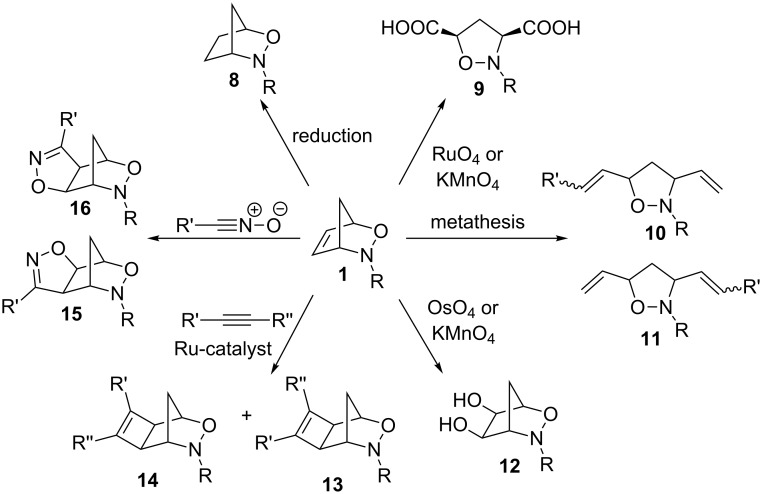
Various reactions involving modification of the alkene component of 3-aza-2-oxabicyclic alkenes.

Heterobicyclic alkenes are useful templates to generate complex cyclic and acyclic systems [1–2]. 3-Aza-2-oxabicyclic alkenes are particularly interesting due to their asymmetric nature and the ability to modify selected components of the molecule to create vastly different products. 3-Aza-2-oxabicyclic alkenes are generally modified in one of four ways (Scheme 1). One of the most interesting manipulations of 3-aza-2-oxabicyclic alkenes is the modification of the alkene component. The manipulation of the olefin can lead to a wide variety of products often in a single step, which is synthetically useful to create many highly substituted products with different stereochemical outcomes (Scheme 2). There are many reported examples in the literature of the modification of the alkene component which includes the reduction to form alkane **8** [3], oxidative cleavage of the C=C bond to form **9** [4], ring-opening metathesis to form functionalized alkenes **10** and **11** [4], dihydroxylation to form diol **12** [5], ruthenium-catalyzed [2 + 2] cycloaddition with unsymmetrical alkynes to form regioisomers **13** and **14** [6], and cycloadditions using nitrile oxides to provide **15** and **16** [7].

**Scheme 3:**
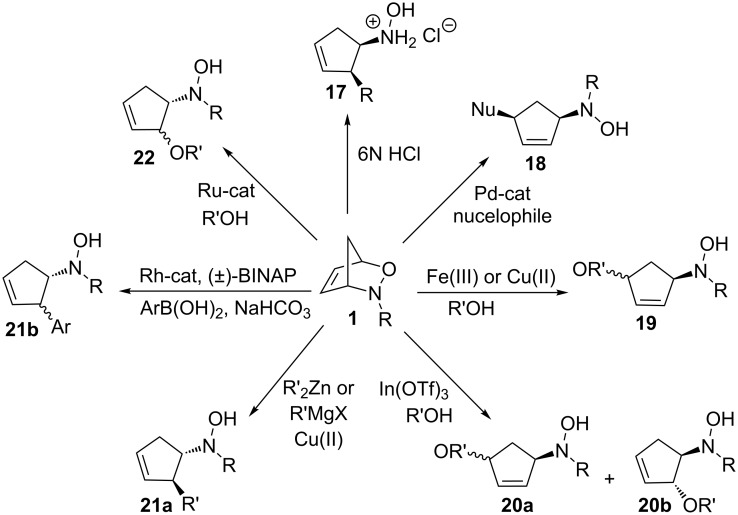
Various reactions involving cleavage of the C–O bond of 3-aza-2-oxabicyclic alkenes.

In the literature, there are also many examples of the cleavage of the C–O bond of 3-aza-2-oxabicyclic alkenes **1** (Scheme 3). This includes the use of protic acid [8], using metal catalysts such as Pd [9], Fe or Cu [10], In [11], organozinc or Grignard reagents [12], Rh [13], and Ru [14] catalysts.

Another interesting modification of the alkene component is cyclopropanation. To date, there are a few reported examples in the literature of the cyclopropanation of 3-aza-2-oxabicyclic alkenes [15–17]. The addition of a cyclopropane unit adds ring-strain to the molecule that could lead to different pathways for ring-opening and further reactivity.

**Scheme 4:**
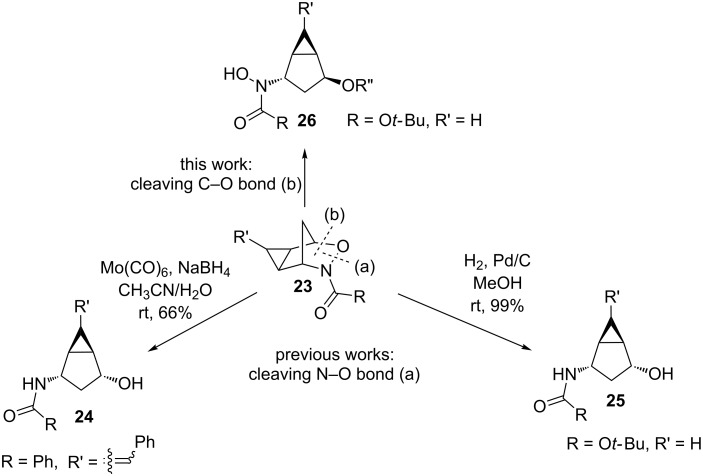
Ring-opening reactions of cyclopropanated 3-aza-2-oxabicyclic alkenes.

**Scheme 5:**
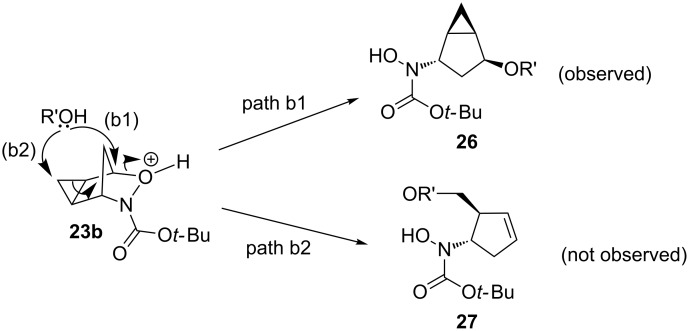
Different possible ring-opening pathways of cyclopropanated 3-aza-2-oxabicyclic alkenes.

While the reactivity of 3-aza-2-oxabicyclo[2.2.1]hept-5-enes has been extensively studied (Scheme 1 and Scheme 2), there are only two examples in the literature investigating the reactivity of the cyclopropanated derivative (Scheme 4) and in both studies, cleavage of the N–O bond (b) was observed. While the Buono group demonstrated the reductive N–O bond cleavage to produce compound **25** as proof-of-principle [16], the Miller group reported the use of the cyclopropanated compound for the synthesis of 2’,3’-methano carbocyclic nucleosides via compound **24** (Scheme 5) [17]. Carbocyclic nucleosides are important synthetic targets because of their use as antiviral and antitumor agents [17]. Replacing the oxygen unit in the parent furanose ring with a methylene unit helps to stabilize the structure against cleavage by nucleoside phosphorylases or hydrolases [18–19]. The addition of a cyclopropane unit could provide further rigidity that could better stabilize the compound, thereby enhancing its biological activity. Both of these reported ring-openings of cyclopropanated 3-aza-2-oxabicyclo[2.2.1]alkenes reductively cleave the N–O bond (a) (Scheme 4), therefore, no examples cleaving the C–O bond have been reported in the literature. In this paper, we aim to explore the use of an acid catalyst with an alcohol nucleophile on the ring-opening of cyclopropanated 3-aza-2-oxabicyclic compound **19** for the cleavage of the C–O bond (b) (Scheme 4). We initially anticipated that the S_N_2’ type ring-opening would occur which would lead to the formation of ring-opened product **27** (Scheme 5). However, in all cases tested, only the S_N_2 type ring-opened product **26** was formed.

## Results and Discussion

A variety of different acid catalysts was screened and the results are summarized in Table 1. In the presence of a Lewis acid catalyst (Table 1, entries 1–3), the reaction did not proceed as seen with FeCl_3_ (Table 1, entry 1) or produced ring-opened product **26** in low yields (Table 1, entries 2 and 3). The effect of inorganic protic acids was then investigated (Table 1, entries 4–6), producing moderate yields of the ring-opened product. The use of fluoroboric acid (Table 1, entry 4) and sulfuric acid (Table 1, entry 5) produced ring-opened product **26** in 45% and 48% yield, respectively, while using nitric acid increased the yield to 56% with trace amount of starting material **23a** recovered. Finally, the effect of organic protic acids was investigated (Table 1, entries 7–9) which produced ring-opened product **26** in low to moderate yields. The use of *p*-toluenesulfonic acid monohydrate produced the ring-opened product at a yield of 38% (Table 1, entry 7) while using camphorsulfonic acid (CSA) increased the yield to 50% but took 46 hours to go to completion (Table 1, entry 8). The organic acid pyridinium *p*-toluenesulfonate (PPTS) produced the highest yield of ring-opened product with a 61% yield (Table 1, entry 9) and was chosen to further optimize reaction conditions.

**Table 1:** Effects of acid catalysts on the ring-opening reaction of cyclopropanated 3-aza-2-oxabicyclic alkene **23a** with alcohols.

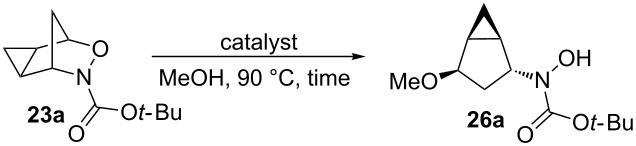			

Entry	Catalyst	Time (h)	Yield (%)^a^

1	FeCl_3_	22	0
2	ZrCl_4_	24	15
3	AlCl_3_	24	32
4	HBF_4_	24	45
5	H_2_SO_4_	24	48
6	HNO_3_	24	56^b^
7	*p*-TsOH·H_2_O	24	38^b^
8	CSA	46	50
9	PPTS	24	61^b^

^a^Isolated yield after column chromatography. ^b^1–4% of starting material was recovered.

A variety of solvents was screened, including polar protic, aromatic, and polar aprotic solvents (Table 2). When methanol was used as the nucleophile and polar protic solvent, the reaction yielded the ring-opened product in a 61% yield (Table 2, entry 1). The use of the aromatic solvent toluene gave a moderate yield of 47% but took 48 hours to go to completion (Table 2, entry 2). Polar aprotic solvents DCE, THF, and 1,4-dioxane were investigated, which produced the ring-opened product **26** in moderate yields (Table 2, entries 3–5). Using DMF decreased the yield significantly to only 6%, with 57% of starting material **23a** recovered after eight days (Table 2, entry 6). The use of the polar aprotic solvent acetonitrile decreased the yield of the reaction to 27% and took almost five days to complete with 3% of starting material **23a** recovered (Table 2, entry 7). Finally, using DMSO decreased the yield to 32% with 5% of starting material recovered after 49 hours (Table 2, entry 8). Since the best result was obtained without the use of a cosolvent, the polar protic nucleophile will be used as both the nucleophile and solvent.

**Table 2:** Effect of solvent on the ring-opening reactions of cyclopropanated 3-aza-2-oxabicyclic alkene **23a** with alcohols.

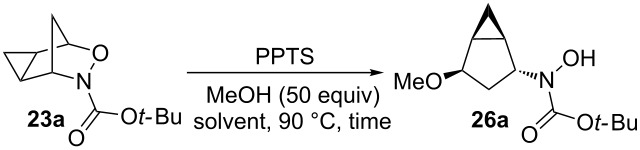				

Entry	Solvent	Time (h)	Yield **26a** (%)^a^	Recovered **23a** (%)^a^

1	MeOH	24	61	4
2	toluene	48	47	0
3	DCE	24	41	0
4	THF	48	39	0
5	1,4-dioxane	24	39	3
6	DMF	190	6	57
7	CH_3_CN	117	27	3
8	DMSO	49	32	5

^a^Isolated yield after column chromatography.

To study the scope of the reaction, the use of different alcohol nucleophiles was tested (Table 3). With a primary alcohol, a decrease in reactivity was seen with increasing chain length (Me < Et < *n*-Bu; Table 3, entries 1–3) while maintaining reasonable yields. When 2-methoxyethanol was used as the nucleophile, the yield was decreased to 42% (Table 3, entry 4) while using isobutyl alcohol produced a similar yield of 41% (Table 3, entry 5). Using 2-methylbutanol produced a 34% yield of a 1:1 diastereomeric ratio of product (Table 3, entry 6) and using allyl alcohol produced ring-opened product **26g** in a 38% yield (Table 3, entry 7). The use of secondary alcohols generally resulted in a decrease of yield of the ring-opened product (Table 3, entries 8–11). Isopropanol achieved a moderate yield of 51% (Table 3, entry 8) while using 2-butanol decreased the yield to 28% of a 1:1 diastereomeric ratio of product (Table 3, entry 9). The cyclic alcohols cyclohexanol and cyclopentanol (Table 3, entries 10 and 11) produced low amounts of the ring-opened alcohol in a 24% and 26% yield, respectively. The use of a tertiary alcohol surprisingly resulted in a moderate yield, with *tert*-butanol producing a 50% yield of product **26l** (Table 3, entry 12, preparation of compound **26l** from **23a** and *t*-BuOH with PPTS was already published in reference [20]). When the aromatic alcohol phenol was investigated as a nucleophile, no reaction occurred though no starting material was recovered (Table 3, entry 13). Although in most cases, the starting material was completely consumed, the yields of these ring-opening reactions were only moderate (26–61%). This may be due to the decomposition or polymerization of the cyclopropanated 3-aza-2-oxabicyclic alkene under the reaction conditions. Through X-ray crystallography [20] and 1D NOESY ^1^H NMR the stereochemistry of the products was confirmed, with the nucleophile added *syn* to the cyclopropane ring and *anti* to the amino alcohol group.

**Table 3:** Scope of the reaction with different alcohol nucleophiles.

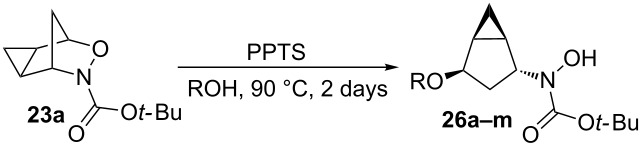			

Entry	ROH	Products	Yield (%)^a^

1	MeOH	**26a**	61
2	EtOH	**26b**	51
3	*n*-BuOH	**26c**	36
4	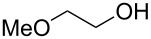	**26d**	42
5	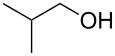	**26e**	41
6	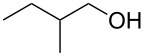	**26f**	34^b^
7	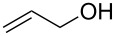	**26g**	38
8	iPrOH	**26h**	51
9	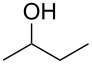	**26i**	28^b^
10	CyOH	**26j**	24
11	cyclopentanol	**26k**	26
12	*t*-BuOH	**26l**	50
13	PhOH	**26m**	0

^a^Isolated yield after column chromatography. ^b^Produced inseparable 1:1 diastereomeric products.

**Scheme 6:**
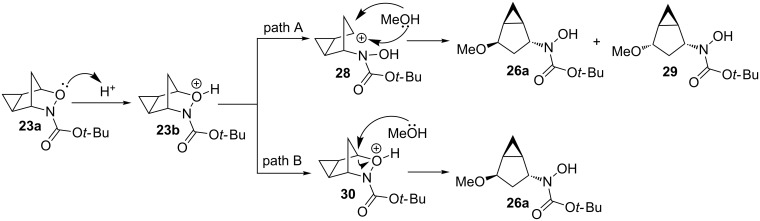
Possible mechanisms for the nucleophilic ring-opening of cyclopropanated 3-aza-2-oxabicyclic alkene **19**.

When forming the ring-opening product, there are two possible mechanisms (Scheme 6).

If the oxygen atom is first protonated followed by cleavage of the C–O bond as seen in path A, the free carbocation **28** would form in an S_N_1-like manner. The nucleophile could therefore attack from either the top or the bottom, forming products **26a** and **29**. Alternatively, in path B the oxygen atom could be protonated and undergo an S_N_2-like mechanism with the nucleophile attacking from the open top face seen in **30**, forming sole product **26a**. If a free carbocation was formed as shown in path A, both stereoisomers **26a** and **29** should have been observed, which was not evident. Also, if a free carbocation was formed the product likely would have undergone rearrangement of the cyclopropyl cation to ring-open the cyclopropane ring and form either a five or six-membered product, however, the cyclopropane is proved to be intact. Therefore, since only one single product **26a** was observed, it is confirmed the product is formed through an S_N_2-like pathway seen in path B.

## Conclusion

In conclusion, we have demonstrated the first examples of acid-catalyzed nucleophilic ring-opening reactions of a cyclopropanated 3-aza-2-oxabicyclic alkene with alcohols. Although this acid-catalyzed ring-opening reaction did not cleave the cyclopropane unit as planned, this represent the first examples of ring-openings of cyclopropanated 3-aza-2-oxabicyclo[2.2.1]alkenes that lead to the cleavage of the C–O bond instead of the N–O bond. Different acid catalysts were tested and it was found that pyridinium toluenesulfonate in methanol gave the best yields in the ring-opening reactions. The scope of the reaction was successfully expanded to include primary, secondary, and tertiary alcohol nucleophiles. Through X-ray crystallography, the stereochemistry of the product was determined which confirmed an S_N_2-like mechanism to form the ring-opened product. Further investigation of the ring-opening reactions of cyclopropanated 3-aza-2-oxabicyclo[2.2.1]alkenes using other metal catalysts, such as those listed in Scheme 3, is ongoing in our laboratory.

## Supporting Information

Experimental procedures and copies of ^1^H and ^13^C NMR spectra for compounds are provided in the Supporting Information.

File 1 Experimental.

File 2 NMR spectra.
