# Supplementary material for: Acid-catalyzed ring-opening reactions of a cyclopropanated 3-aza-2-oxabicyclo[2.2.1]hept-5-ene with alcohols
Source: Beilstein J Org Chem. 2017 Dec 27;13:2888–94. doi: 10.3762/bjoc.13.281 (PMC5753073; doi:10.3762/bjoc.13.281)
Supplement: File 1 — Experimental. [file Beilstein_J_Org_Chem-13-2888-s001.pdf]

Supporting Information File 1

for

Acid-catalyzed ring-opening reactions of a cyclopropanated 3-aza-2-oxabicyclo[2.2.1]hept-5-ene with alcohols

Katrina Tait[1], Alysia Horvath[1], Nicolas Blanchard[2] and William Tam[1]*

Address: [1]Guelph-Waterloo Centre for Graduate Work in Chemistry and Biochemistry, Department of Chemistry, University of Guelph, Guelph, Ontario, N1G 2W1, Canada and [2]Laboratoire de Chimie Moléculaire, ECPM-CNRS UMR7509, University of Strasbourg, 25 rue Becquerel, 67087 Strasbourg, France

Email: William Tam - wtam@uoguelph.ca

* Corresponding author

Experimental

Table of Contents

General considerations S2

Experimental procedures and full characterization for key compound **23a** S2

General procedure for acid-catalyzed ring-opening reaction of cyclopropanated 3-aza-2-oxabicyclo[2.2.1]hept-5-enes with alcohols S3

Characterization data for compounds **26a–l** S3–S8

General considerations

All ring-opening reactions were carried out under inert atmospheric conditions. All glassware was oven dried overnight before use. Commercial reagents were all used as received from their respective suppliers. Flash column chromatography was performed on 230–400 mesh silica gel purchased from Silicycle. Analytical TLC was performed on pre-coated silica gel 250 μm 60 F254 aluminum plates purchased from Silicycle. TLC visualization was carried out under UV light and *p*-anisaldehyde stain. Infrared samples were acquired as solids or as neat oils on a Bruker ALPHA platinum single reflection diamond ATR spectrophotometer and are reported in wave numbers (cm^{-1}). ^{1}H and ^{13}C NMR spectra were recorded on a Bruker Avance 400 MHz spectrometer ($CDCl_3$: δ 7.24 ppm (^{1}H at 400 MHz) or δ 77.0 ppm (^{13}C at 100 MHz)). HRMS analyses were performed at the Queen's Mass Spectrometry and Proteomics Unit, Kingston, Ontario. The samples were ionized by electrospray (ESI) and detection of the ions was performed by time of flight (TOF).

Experimental procedures and full characterization data for previously reported key compounds

For the complete experimental procedure and full characterization data of starting material **23a** see our previous report on the synthesis of cyclopropanated 3-aza-2-oxabicyclic alkenes [1].

General procedure for the acid-catalyzed ring-opening reaction of cyclopropanated 3-aza-2-oxabicyclo[2.2.1]hept-5-enes with alcohols

In a similar manner as described before (reference [2]), in a small screw-cap vial containing a stir-bar under an inert atmosphere, catalyst was added (0.1 equiv). Cyclopropanated 3-aza-2-oxabicyclo[2.2.1]hept-5-ene (1.0 equiv) was dissolved in alcohol (0.5 mL) and added to the reaction. The vial was sealed and secured tightly with polytetrafluoroethylene (PTFE) thread-seal tape and paraffin film. The reaction was heated to 90 °C with continuous stirring for 1–8 days. The crude product was directly loaded onto a chromatography column and purified (EtOAC:hexanes).

***tert*-Butyl hydroxy((1*R**,2*R**,4*R**,5*S**)-4-methoxybicyclo[3.1.0]hexan-2-yl)carbamate, 26a (Table 1, entry 9):** Yield: 61% (23.4 mg, 0.096 mmol); white solid; m.p. 89-92°C; R_f = 0.35 (EtOAc-hexanes, 1:1); IR (ν, cm^{-1}): 3223, 2975, 2829, 1687, 1451, 1364, 1304, 1249, 1163, 1107, 1076, 974. ^1H NMR ($CDCl_3$, 400 MHz) δ: 7.03 (br.s, 1H, OH), 4.45 (d, 1H, J = 7.6 Hz), 4.43-4.39 (m, 1H), 3.32 (s, 3H, CH_3), 1.98 (dd, J = 14.6 and 8.2 Hz, 1H), 1.76-1.71 (m, 1H), 1.45 (s, 10H), 1.35-1.31 (m, 1H), 0.53-0.48 (m, 1H), 0.41-0.37 (m, 1H); ^{13}C NMR (100 MHz, $CDCl_3$) δ: 156.9, 82.2, 82.1, 60.4, 57.0, 33.0, 28.3, 20.6, 20.5, 4.2; HRMS (ESI) calcd. for $C_{12}H_{21}NNaO_4$ $(M+Na)^+$: 266.1363; found 266.1365.

***tert*-Butyl hydroxy((1*R**,2*R**,4*R**,5*S**)-4-ethoxybicyclo[3.1.0]hexan-2-yl)carbamate, 26b (Table 3, entry 2):** Yield: 51% (18.7 mg, 0.072 mmol); white solid; m.p. 90-92°C; R_f= 0.51 (EtOAc-hexanes, 1:1); IR (ν, cm^{-1}): 3276, 2979, 1691, 1451, 1364, 1332, 1305, 1246, 1166,

1104, 1077, 1009, 945. ^{1}H NMR ($CDCl_3$, 400 MHz) δ: 7.01-6.83 (br. m, 1H), 4.54-4.49 (m, 1H), 4.45 (d, J = 7.6 Hz, 1H), 3.59-3.52 (m, 1H), 3.46-3.39 (m, 1H), 1.97 (dd, J = 14.5 and 8.0 Hz, 1H), 1.74-1.69 (m, 1H), 1.48-1.41 (m, 10H), 1.34-1.29 (m, 1H), 1.18 (t, J = 7.0 Hz, 1H), 0.53-0.48 (m, 1H), 0.43-0.40 (m, 1H); ^{13}C NMR (100 MHz, $CDCl_3$) δ: 156.9, 82.0, 80.4, 64.7, 60.3, 33.3, 28.3, 20.9, 20.5, 15.6, 4.3; HRMS (ESI) calcd. for $C_{13}H_{23}NNaO_4$ $(M+Na)^+$: 280.1519; found 280.1507.

***tert*-Butyl hydroxy((1*R**,2*R**,4*R**,5*S**)-4-butoxybicyclo[3.1.0]hexan-2-yl)carbamate, 26c (Table 3, entry 3):** Yield: 36% (15.5 mg, 0.054 mmol); clear oil; R_f = 0.59 (EtOAc-hexanes, 1:1); IR (ν, cm^{-1}): 3238, 2931, 2872, 1685, 1457, 1392,1366, 1333, 1255, 1168, 1101, 1009, 914; ^{1}H NMR ($CDCl_3$, 400 MHz) δ: 7.04 (br.s, 1H), 4.52-4.46 (m, 1H), 4.44 (d, J = 7.6 Hz, 1H), 3.51-3.46 (m, 1H), 3.38-3.33 (m, 1H), 1.97 (dd, J = 14.5 and 8.1 Hz, 1H), 1.74-1.69 (m, 1H), 1.54-1.49 (m, 2H), 1.48-1.42 (br. m, 10H), 1.36-1.29 (m, 3H), 0.88 (t, J = 7.4 Hz, 3H), 0.52-0.47 (m, 1H), 0.42-0.39 (m, 1H); ^{13}C NMR (100 MHz, $CDCl_3$) δ: 156.9, 82.0, 80.4, 69.2, 60.32, 33.3, 32.1, 28.4, 20.9, 20.5, 19.4, 13.9, 4.3; HRMS (ESI) calcd. for $C_{15}H_{27}NNaO_4$ $(M+Na)^+$: 308.1832; found 308.1831.

***tert*-Butylhydroxy((1*R**,2*R**,4*R**,5*S**)-4--(2-Methoxyethoxy)bicyclo[3.1.0]hexan-2-yl)carbamate , 26d (Table 3, entry 4):** Yield: 42% (17.3 mg, 0.060 mmol); clear oil; R_f = 0.20 (EtOAc-hexanes 1:1); IR (ν, cm^{-1}): 3461, 3056, 2952, 1932, 1492, 1446, 1248, 1156, 1048, 836; ^{1}H NMR ($CDCl_3$, 400 MHz) δ: 7.02 (br.s, 1H), 4.56-4.51 (m, 1H), 4.44 (d, J = 7.6 Hz, 1H), 3.69-3.63 (m, 1H), 3.56-3.50 (m, 3H), 3.35 (s, 3H), 1.98 (dd, J = 14.5 and 8.0 Hz, 1H), 1.75-1.69 (m, 1H), 1.53-1.45 (m, 10H), 1.34-1.30 (m, 1H), 0.53-0.48 (m, 1H), 0.45-0.42 (m, 1H); ^{13}C NMR

(100 MHz, $CDCl_3$) δ: 156.9, 82.0, 81.1, 72.1, 68.5, 60.3, 59.0, 33.2, 28.3, 20.7, 20.7, 4.3; HRMS (ESI) calcd. for $C_{14}H_{25}NNaO_5$ $(M+Na)^+$: 310.1625; found 310.1619.

***tert*-Butyl hydroxy((1*R**,2*R**,4*R**,5*S**)-4-isobutoxybicyclo[3.1.0]hexan-2-yl)carbamate, 26e (Table 3, entry 5):** Yield: 41% (16.5 mg, 0.058 mmol); clear oil; R_f = 0.65 (EtOAc-hexanes, 1:1); IR (ν, cm^{-1}): 3231, 2955, 1686, 1457, 1366, 1332, 1254, 1168, 1100, 1009, 985; ^1H NMR ($CDCl_3$, 400 MHz) δ: 6.57 (br.s, 1H), 4.50-4.44 (m, 2H), 3.27-3.23 (m, 1H), 3.14-3.10 (m, 1H), 1.95 (dd, J = 14.5 and 8.0 Hz, 1H), 1.82 (sept, J = 6.7 Hz, 1H), 1.74-1.69 (m, 1H), 1.49-1.41 (m, 10H), 1.32-1.30 (m, 1H), 0.87 (dd, J = 6.7 and 0.4 Hz, 6H), 0.52-0.47 (m, 1H), 0.43-0.39 (m, 1H); ^{13}C NMR (100 MHz, $CDCl_3$) δ: 156.9, 82.1, 80.5, 76.5, 60.3, 33.3, 28.6, 28.3, 20.9, 20.5, 19.6, 19.5, 4.3; HRMS (ESI) calcd. for $C_{15}H_{27}NNaO_4$ $(M+Na)^+$: 308.1832; found 308.1831.

***tert*-Butylhydroxy((1*R**,2*R**,4*R**,5*S**)-4-(2-methylbutoxy)bicyclo[3.1.0]hexan-2-yl)carbamate, 26f (Table 3, entry 6):** Yield: 34% (15.0 mg, 0.050 mmol; inseparable 1:1 mixture of diastereomers); clear oil; R_f = 0.67 (EtOAc-hexanes, 1:1); IR (ν, cm^{-1}): 3265, 2962, 1686, 1457, 1392, 1366, 1333, 1255, 1168, 1100, 1009, 916; ^1H NMR ($CDCl_3$, 400 MHz) δ: 6.85 (br.s, 1H), 4.49-4.43 (m, 2H), 3.36-3.09 (m, 2H), 1.95 (dd, J = 14.5 and 8.0 Hz, 1H), 1.73-1.68 (m, 1H), 1.61-1.59 (m, 1H), 1.47-1.39 (m, 11H), 1.33-1.28 (m, 1H), 1.09-1.06 (m, 1H), 0.87-0.84 (m, 6H), 0.52-0.46 (m, 1H), 0.42-0.39 (m, 1H); ^{13}C NMR (100 MHz, $CDCl_3$) δ: 156.9, 82.0, 80.5, 80.5, 74.9, 74.8, 60.3, 35.1, 35.0, 33.3, 28.3, 26.4, 26.3, 20.9, 20.8, 20.6, 20.5, 16.7, 16.6, 11.3, 11.2, 4.3, 4.2; HRMS (ESI) calcd. for $C_{16}H_{29}NNaO_4$ $(M+Na)^+$: 322.1989; found 322.1979.

***tert*-Butyl hydroxy((1*R**,2*R**,4*R**,5*S**)-4-allyloxybicyclo[3.1.0]hexan-2-yl)carbamate, 26g (Table 3, entry 7):** Yield: 38% (14.8 mg, 0.055 mmol); clear oil; R_f = 0.44 (EtOAc-hexanes 1:1); IR (ν, cm^{-1}): 3249, 3076, 2978, 1684, 1456, 1392, 1366, 1331, 1254, 1167, 1084, 1011, 920; ^{1}H NMR ($CDCl_3$, 400 MHz) δ: 6.79 (br.s, 1H), 5.96-5.87 (m, 1H), 5.28-5.22 (m, 1H), 5.15-5.11 (m, 1H), 4.57-4.52 (m, 1H), 4.45 (d, J = 7.6 Hz, 1H), 4.06-4.01 (m, 1H), 3.96-3.91 (m, 1H), 1.97 (dd, J = 14.6 and 8.1 Hz, 1H), 1.51-1.44 (m, 10H), 1.34-1.30 (m, 1H), 0.54-0.49 (m, 1H), 0.45-0.42 (m, 1H); ^{13}C NMR (100 MHz, $CDCl_3$) δ: 156.9, 135.3, 116.8, 82.1, 80.3, 70.6, 60.3, 33.3, 28.3, 20.8, 20.7, 4.3; HRMS (ESI) calcd. for $C_{14}H_{23}NNaO_4$ $(M+Na)^+$: 292.1514; found 292.1519.

***tert*-Butyl hydroxy((1*R**,2*R**,4*R**,5*S**)-4-isopropoxybicyclo[3.1.0]hexan-2-yl)carbamate, 26h (Table 3, entry 8):** Yield: 51% (20.0 mg, 0.074 mmol); white solid; m.p. 68-70°C; R_f = 0.59 (EtOAc-hexanes 1:1); IR (ν, cm^{-1}): 3248, 2971, 1685, 1456, 1366, 1328, 1254, 1167, 1113, 1068, 1011, 956; ^{1}H NMR ($CDCl_3$, 400 MHz) δ: 7.12-7.03 (br.m, 1H), 4.63-4.58 (m, 1H), 4.43 (d, J = 7.6 Hz, 1H), 3.65 (sept, J = 6.1 Hz, 1H), 1.93 (dd, J = 14.5 and 8.1 MHz, 1H), 1.70-1.65 (m, 1H), 1.45-1.38 (m, 10H), 1.31-1.27 (m, 1H), 1.13 (dd, J = 14.4 and 6.2 HZ, 6H), 0.52-0.47 (m, 1H), 0.43-0.40 (m, 1H); ^{13}C NMR (100 MHz, $CDCl_3$) δ: 156.9, 82.0, 77.9, 69.9, 60.3, 33.7, 28.3, 22.9, 22.5, 21.4, 20.3, 4.3; HRMS (ESI) calcd. for $C_{14}H_{25}NNaO_4$ $(M+Na)^+$: 294.1676; found 294.1669.

***tert*-Butyl hydroxy((1*R**,2*R**,4*R**,5*S**)-4-*sec*-butoxybicyclo[3.1.0]hexan-2-yl)carbamate, 26i (Table 3, entry 9):** Yield: 28% (11.3 mg, 0.040 mmol, inseparable 1:1 mixture of diastereomers); clear oil; R_f = 0.57 (EtOAc-hexanes 1:1); IR (ν, cm^{-1}): 3238, 2970, 2930, 1685,

1456, 1366, 1330, 1255, 1168, 1110, 1070, 1010, 997; ^{1}H NMR ($CDCl_3$, 400 MHz) δ: 6.95 (br.s, 1H), 4.64-4.57 (m, 1H), 4.43 (d, J = 7.6 Hz, 1H), 3.44-3.32 (m, 1H), 1.96-1.90 (m, 1H), 1.69-1.65 (m, 1H), 1.52-1.48 (m, 1H), 1.47-1.44 (m, 10H), 1.42-1.37 (m, 1H), 1.32-1.27 (m, 1H), 1.10 (dd, J = 17.1 and 6.1 Hz, 3H), 0.91-0.83 (m, 3H), 0.51-0.41 (m, 2H); ^{13}C NMR (100 MHz, $CDCl_3$) δ:156.9, 156.9, 82.0, 78.3, 77.9, 75.5, 75.2, 60.3, 60.2, 33.7, 33.4, 29.9, 29.6, 28.3, 21.6, 21.1, 20.4, 20.3, 19.9, 10.2, 10.1, 4.4, 4.3; HRMS (ESI) calcd. for $C_{15}H_{27}NNaO_4$ $(M+Na)^+$: 308.1832; found 320.1825.

***tert*-Butyl hydroxy((1*R**,2*R**,4*R**,5*S**)-4-cyclohexyloxybicyclo[3.1.0]hexan-2-yl)carbamate, 26j (Table 3, entry 10):** Yield: 24% (11.1 mg, 0.036 mmol); white solid; m.p. 89-92°C; R_f = 0.63 (EtOAc-hexanes 1:1); IR (ν, cm^{-1}): 3273, 2931, 2852, 1712, 1450, 1360, 1306, 1255, 1170, 1115, 1068, 1018, 963; ^{1}H NMR ($CDCl_3$, 400 MHz) δ: 6.82 (br.s, 1H), 4.67-4.62 (m, 1H), 4.43 (d, J = 7.6 Hz, 1H), 3.31-3.24 (m, 1H), 1.97-1.89 (m, 3H), 1.72-1.64 (m, 3H), 1.53-1.48 (m, 1H), 1.47-1.39 (m, 10H), 1.30-1.12 (m, 6H), 0.51-0.46 (m, 1H), 0.44-0.41(m, 1H); ^{13}C NMR (100 MHz, $CDCl_3$) δ: 156.9, 82.1, 77.7, 76.7, 60.3, 33.8, 33.3, 32.9, 28.4, 25.8, 24.6, 24.6, 21.5, 20.2, 4.3; HRMS (ESI) calcd. for $C_{17}H_{29}NNaO_4$ $(M+Na)^+$: 334.1989; found 334.1981.

***tert*-Butylhydroxy((1*R**,2*R**,4*R**,5*S**)-4-cyclopentyloxybicyclo[3.1.0]hexan-2-yl)carbamate, 26k (Table 3, entry 11):** Yield: 26% (11.6 mg, 0.039 mmol); clear oil; R_f = 0.60 (EtOAc-hexanes 1:1); IR (ν, cm^{-1}): 3244, 2961, 1684, 1455, 1392, 1366, 1333, 1254, 1168, 1083, 1009, 915; ^{1}H NMR ($CDCl_3$, 400 MHz) δ: 6.81 (br.s, 1H), 4.57-4.52 (m, 1H), 4.42 (d, J = 7.6 Hz, 1H), 3.99-3.94 (m, 1H), 1.93 (dd, J = 14.5 and 8.0 Hz, 1H), 1.78-1.60 (m, 7H), 1.49-1.43 (m, 12H), 1.32-1.27 (m, 1H), 0.51-0.49 (m, 1H), 0.43-0.39 (m, 1H); ^{13}C NMR (100 MHz, $CDCl_3$) δ: 156.9,

82.1, 80.1, 78.8, 60.2, 33.5, 32.9, 32.5, 28.3, 23.5, 23.4, 21.3, 20.4, 4.4; HRMS (ESI) calcd. for $C_{16}H_{27}NNaO_4$ $(M+Na)^+$: 320.1832; found 320.1826.

***tert*-Butylhydroxy((1*R**,2*R**,4*R**,5*S**)-4-*tert*-Butoxybicyclo[3.1.0]hexan-2-yl)carbamate, 26l (Table 3, entry 12):** Preparation of compound **26l** from **23a** and t-BuOH with PPTS was already published in reference [2]. Yield: 50% (23.5 mg, 0.082 mmol); white solid; m.p. 96-99°C; R_f = 0.59 (EtOAc-hexanes 1:1); IR (ν, cm^{-1}): 3302, 2979, 1697, 1390, 1364, 1338, 1311, 1245, 1173, 1109, 1080, 1054, 1029, 948; ^1H NMR ($CDCl_3$, 400 MHz) δ: 7.13 (br.s, 1H), 4.68-4.63 (m, 1H), 4.39 (d, J = 7.6 Hz, 1H), 1.83 (dd, J = 14.5 and 8.2 Hz, 1H), 1.59-1.53 (m, 1H), 1.48-1.37 (m, 10H), 1.25-1.15 (m, 10H), 0.52-0.46 (m, 1H), 0.44-0.41 (m, 1H); ^{13}C NMR (100 MHz, $CDCl_3$) δ: 156.9, 81.9, 73.2, 72.9, 60.1, 34.9, 28.6, 28.4, 23.0, 19.7, 4.3; HRMS (ESI) calcd. for $C_{15}H_{27}NNaO_4$ $(M+Na)^+$: 308.1832; found 308.1825.

References:

[1] Carlson, E.; Duret, G.; Blanchard, N.; Tam, W. *Synth. Commun.* **2015**, *46*, 55-62. doi: 00397911.2015.1118124.

[2] Lough, A. J.; Tait, K.; Horvath, A.; Tam, W. *IUCrData* **2017**, *2,* x171419. doi: 10.1107/*S*2414314617014195
