# Supplementary material for: Acid-catalyzed ring-opening reactions of a cyclopropanated 3-aza-2-oxabicyclo[2.2.1]hept-5-ene with alcohols
Source: Beilstein J Org Chem. 2017 Dec 27;13:2888–94. doi: 10.3762/bjoc.13.281 (PMC5753073; doi:10.3762/bjoc.13.281)
Supplement: File 2 — NMR spectra. [file Beilstein_J_Org_Chem-13-2888-s002.pdf]

Supporting Information File 2

for

Acid-catalyzed ring-opening reactions of a cyclopropanated 3-aza-2-oxabicyclo[2.2.1]hept-5-ene with alcohols

Katrina Tait[1], Alysia Horvath[1], Nicolas Blanchard[2] and William Tam[1]*

Address: [1]Guelph-Waterloo Centre for Graduate Work in Chemistry and Biochemistry, Department of Chemistry, University of Guelph, Guelph, Ontario, N1G 2W1, Canada and [2]Laboratoire de Chimie Moléculaire, ECPM-CNRS UMR7509, University of Strasbourg, 25 rue Becquerel, 67087 Strasbourg, France

Email: William Tam - wtam@uoguelph.ca

* Corresponding author

NMR spectra

Table of Contents

^{1}H and ^{13}C NMR spectra for new compounds S2–S13

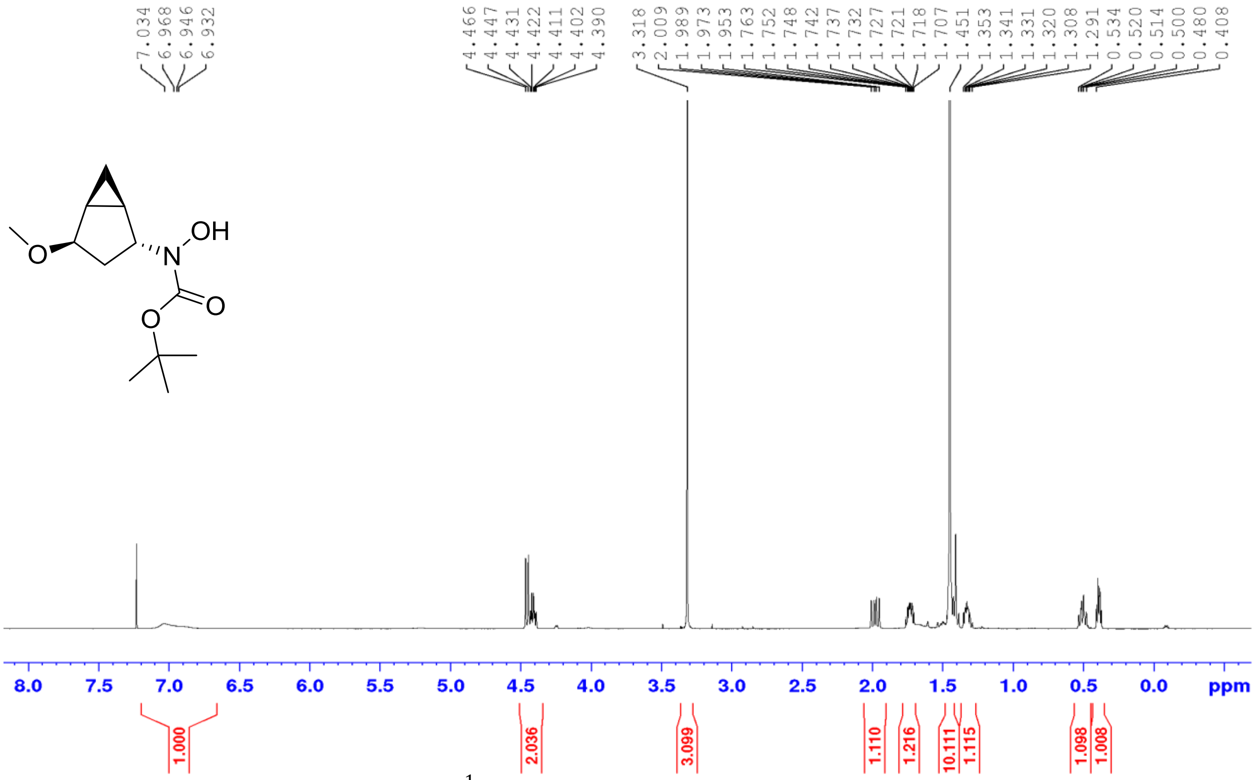

400 MHz ^{1}H NMR spectrum of **26a** in $CDCl_3$

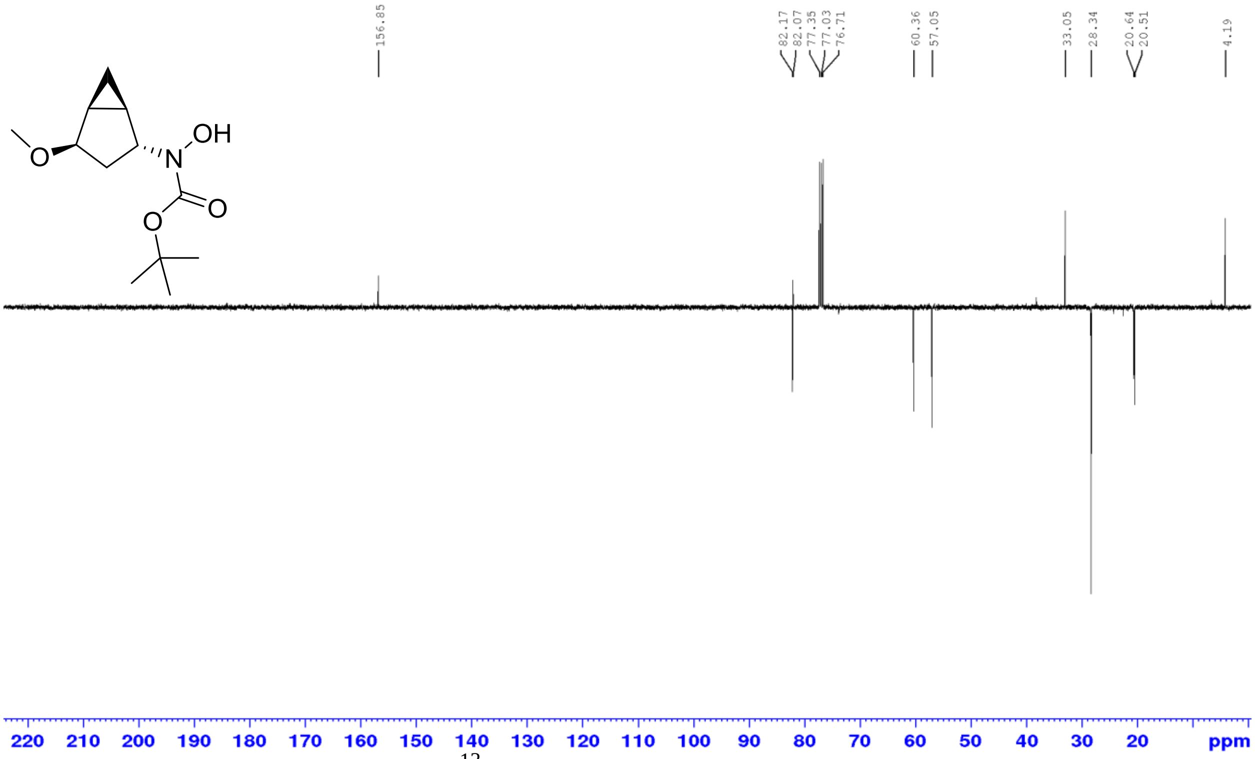

100 MHz ^{13}C NMR spectrum of **26a** in $CDCl_3$

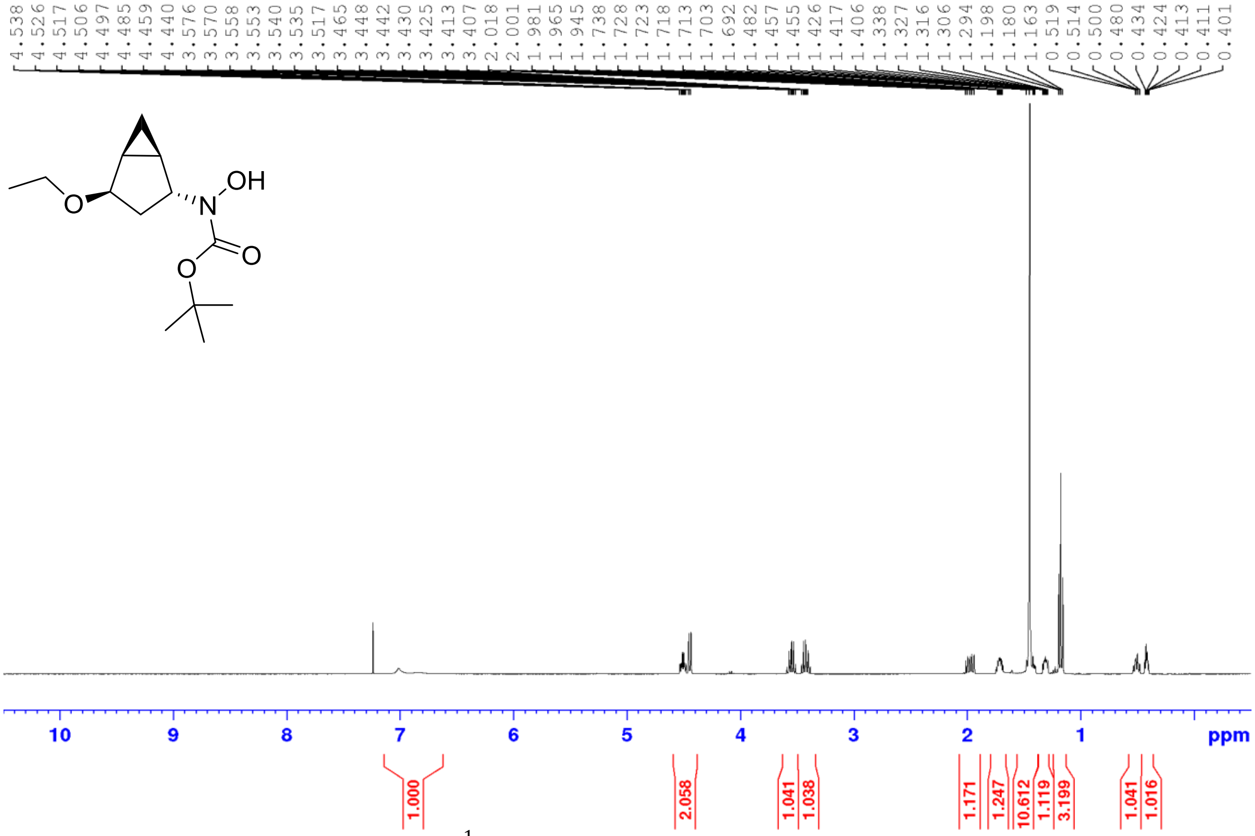

400 MHz ^{1}H NMR spectrum of **26b** in $CDCl_3$

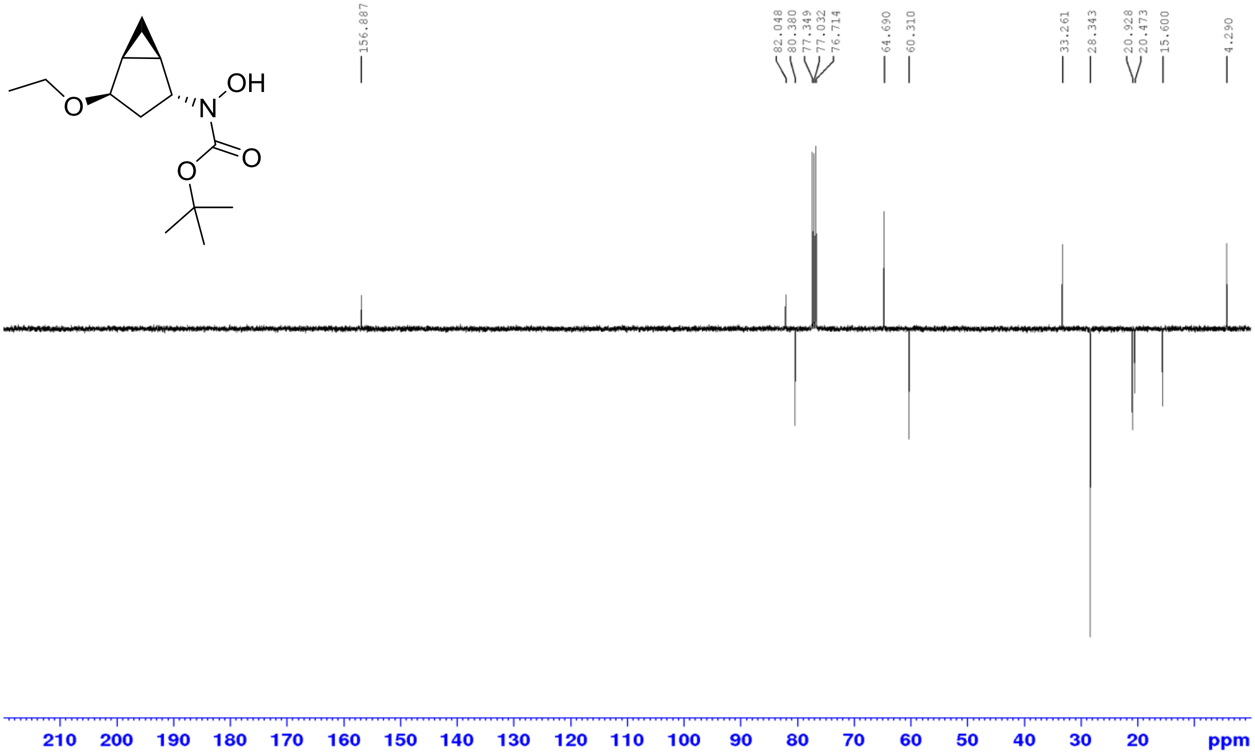

100 MHz ^{13}C NMR spectrum of **26b** in $CDCl_3$

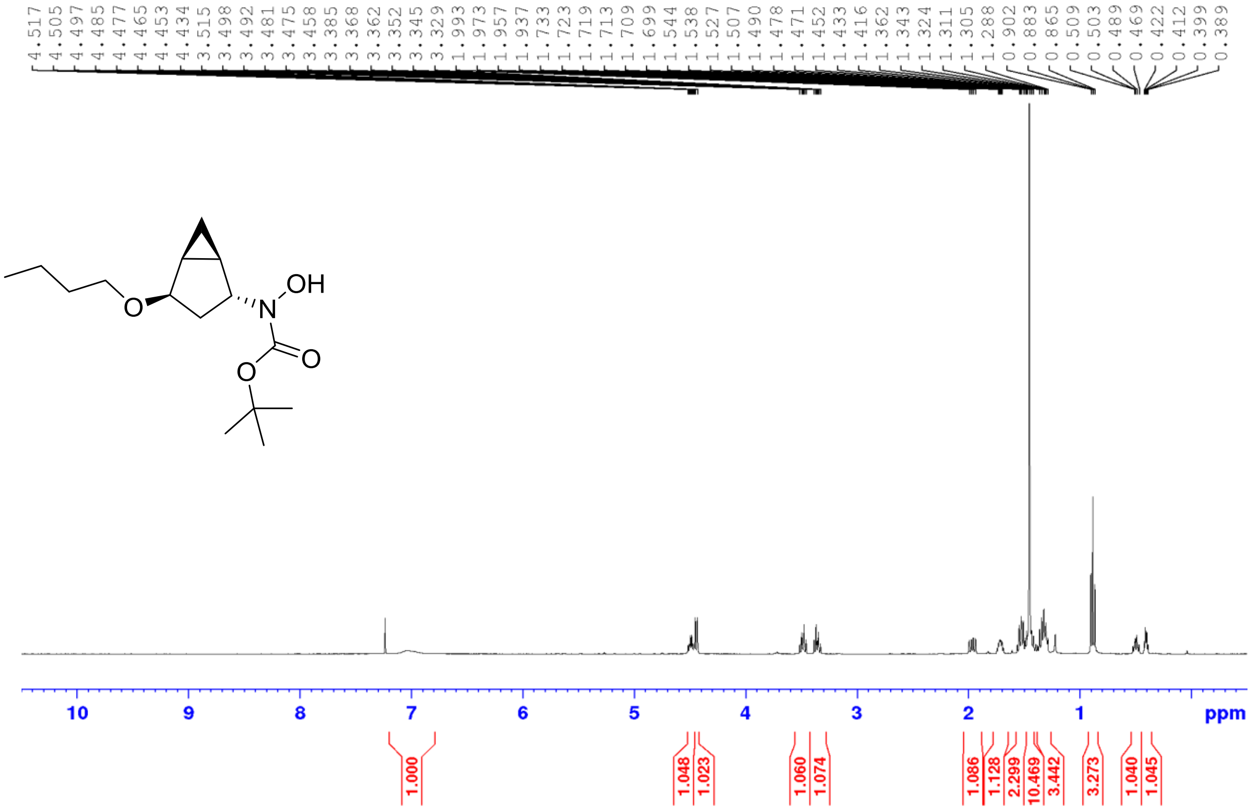

400 MHz ^{1}H NMR spectrum of **26c** in $CDCl_3$

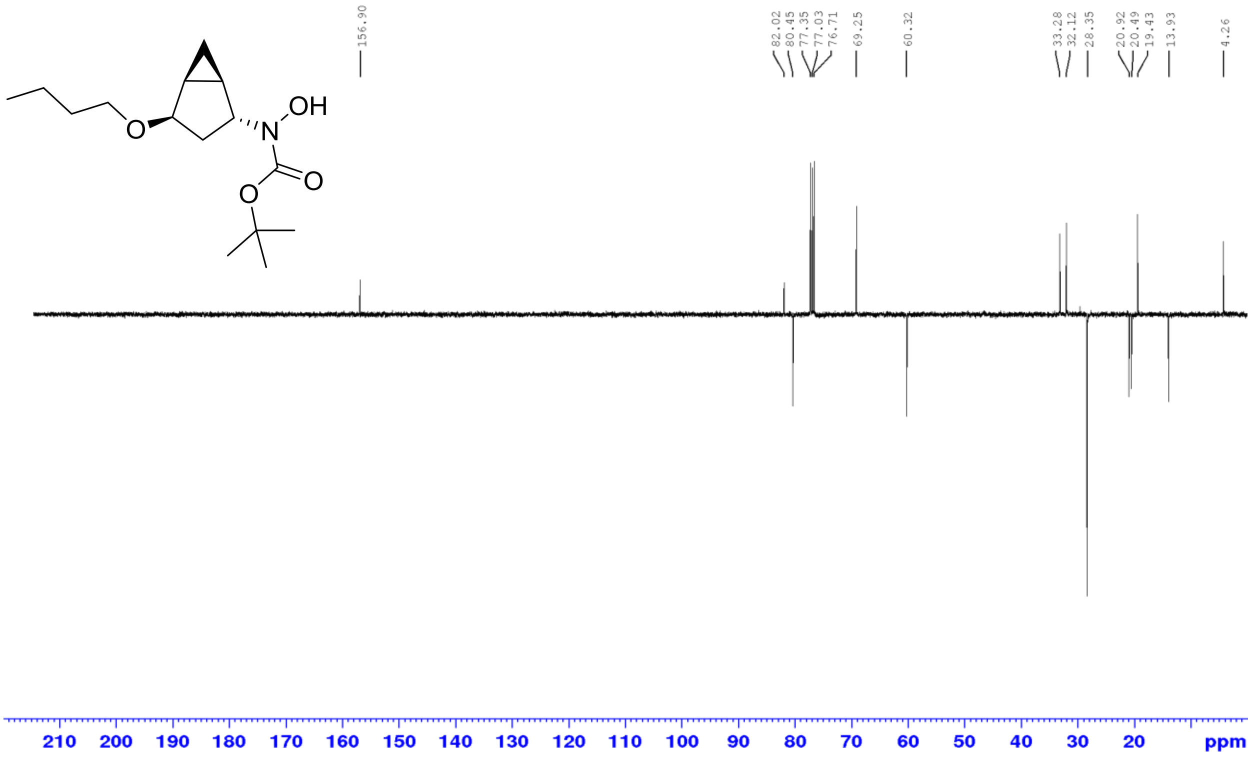

100 MHz ^{13}C NMR spectrum of **26c** in $CDCl_3$

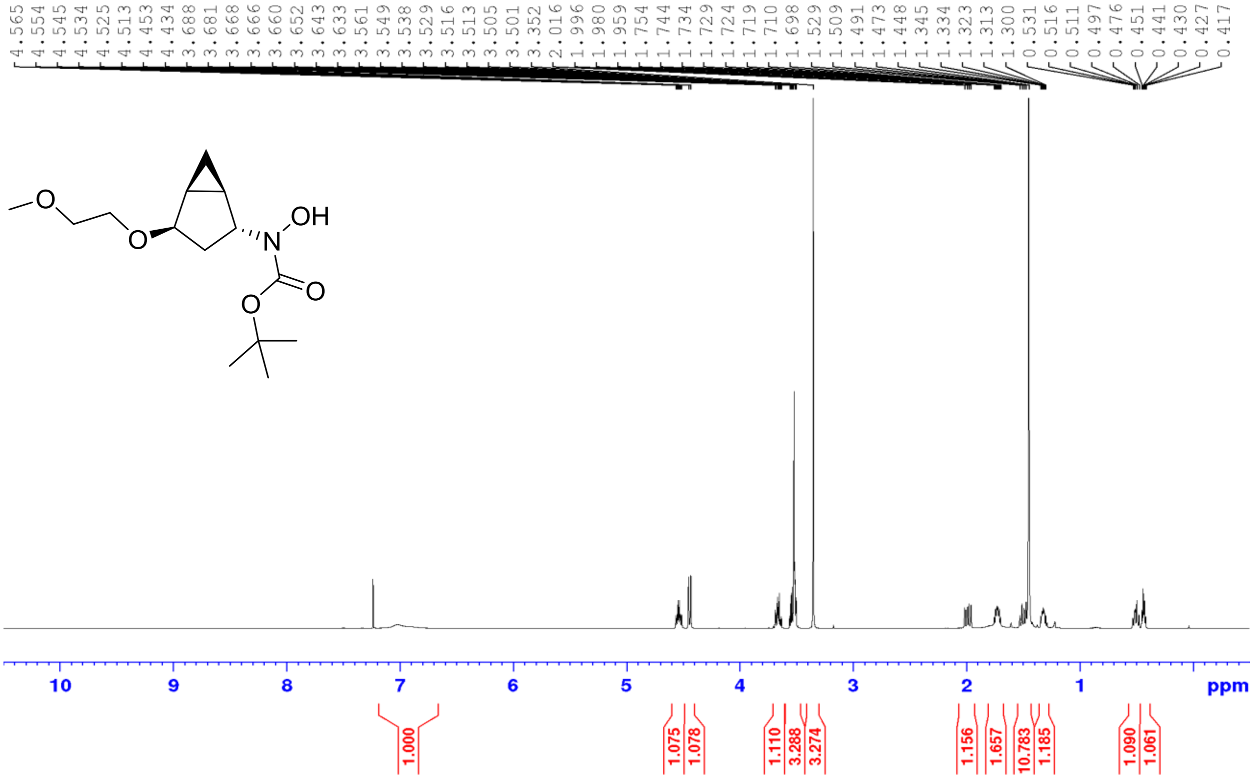

400 MHz ^{1}H NMR spectrum of **26d** in $CDCl_3$

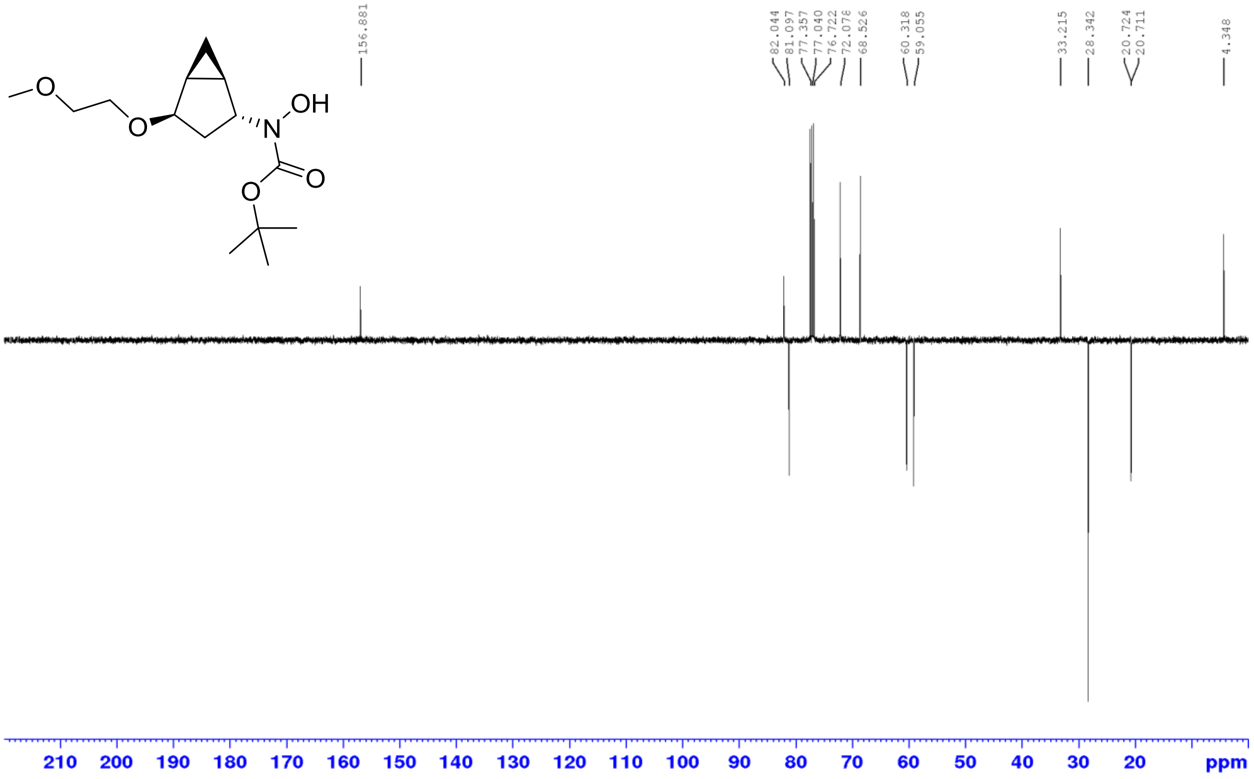

100 MHz ^{13}C NMR spectrum of **26d** in $CDCl_3$

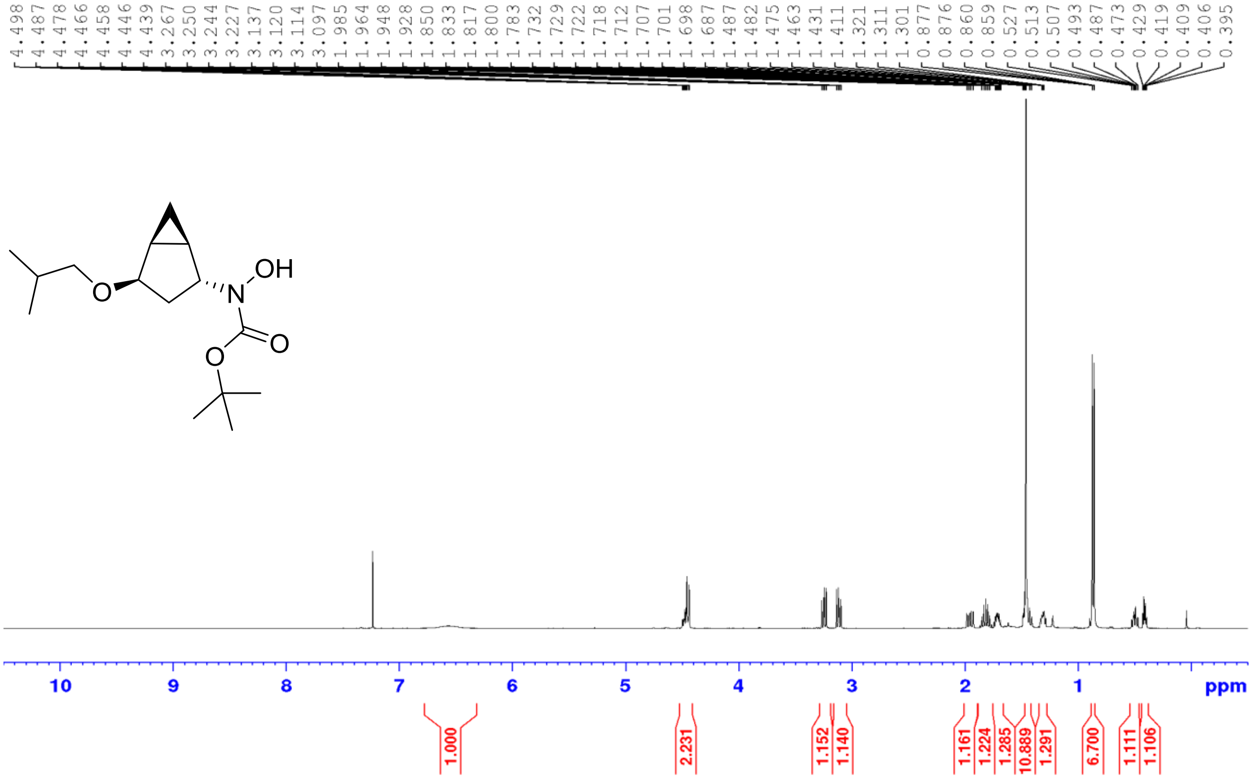

400 MHz ^{1}H NMR spectrum of **26e** in $CDCl_3$

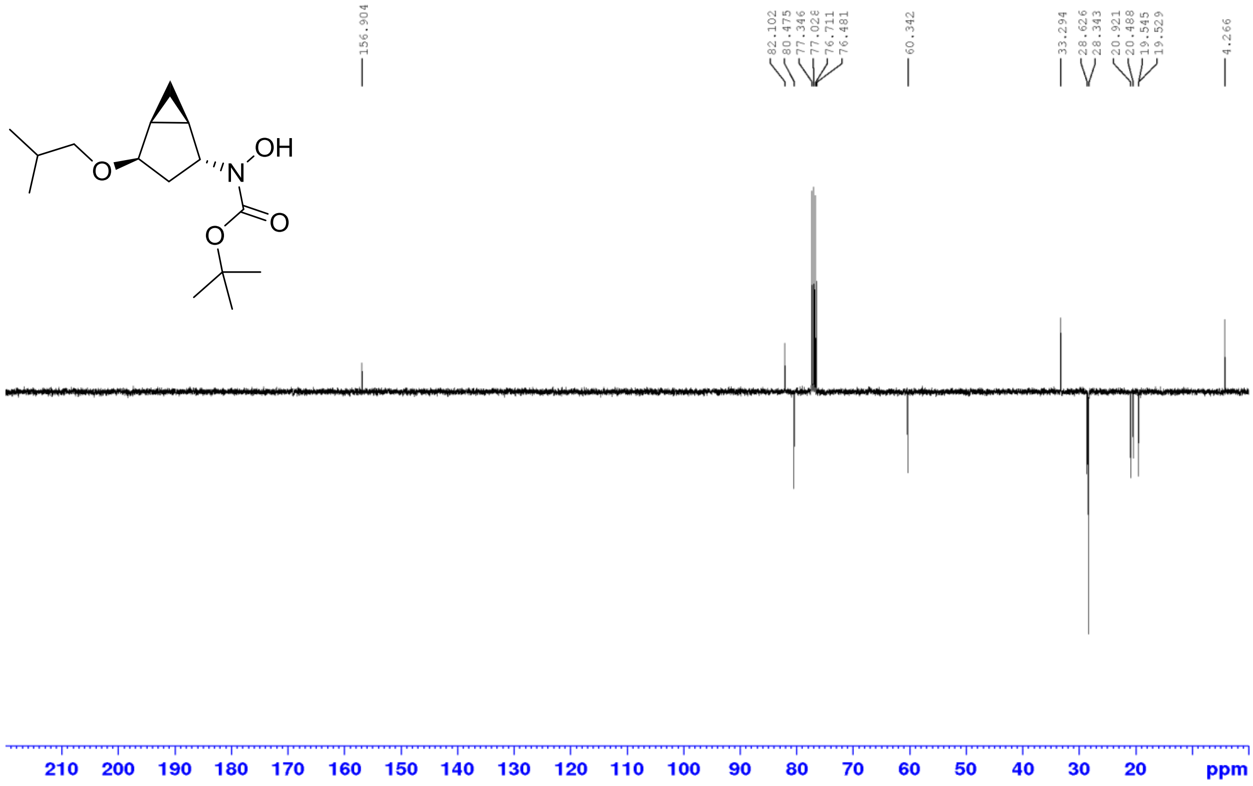

100 MHz ^{13}C NMR spectrum of **26e** in $CDCl_3$

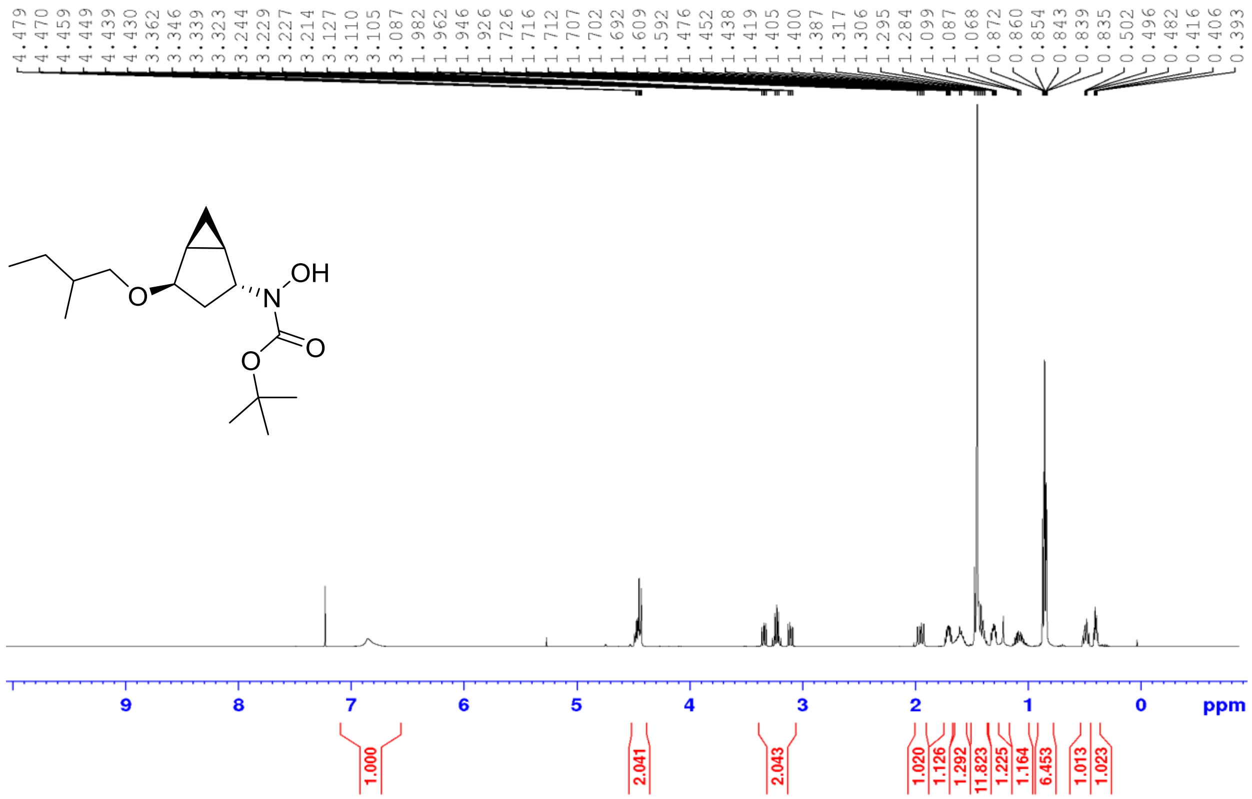

400 MHz ^{1}H NMR spectrum of **26f** in $CDCl_3$

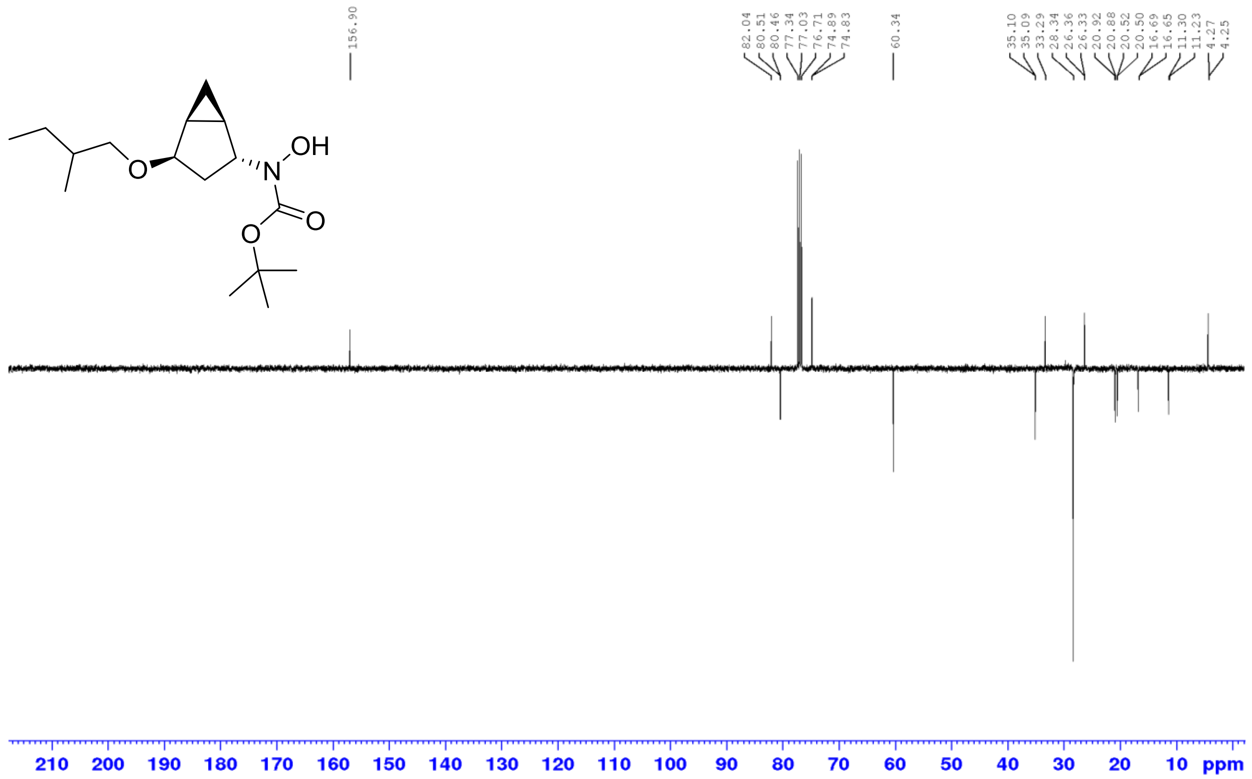

100 MHz ^{13}C NMR spectrum of **26f** in $CDCl_3$

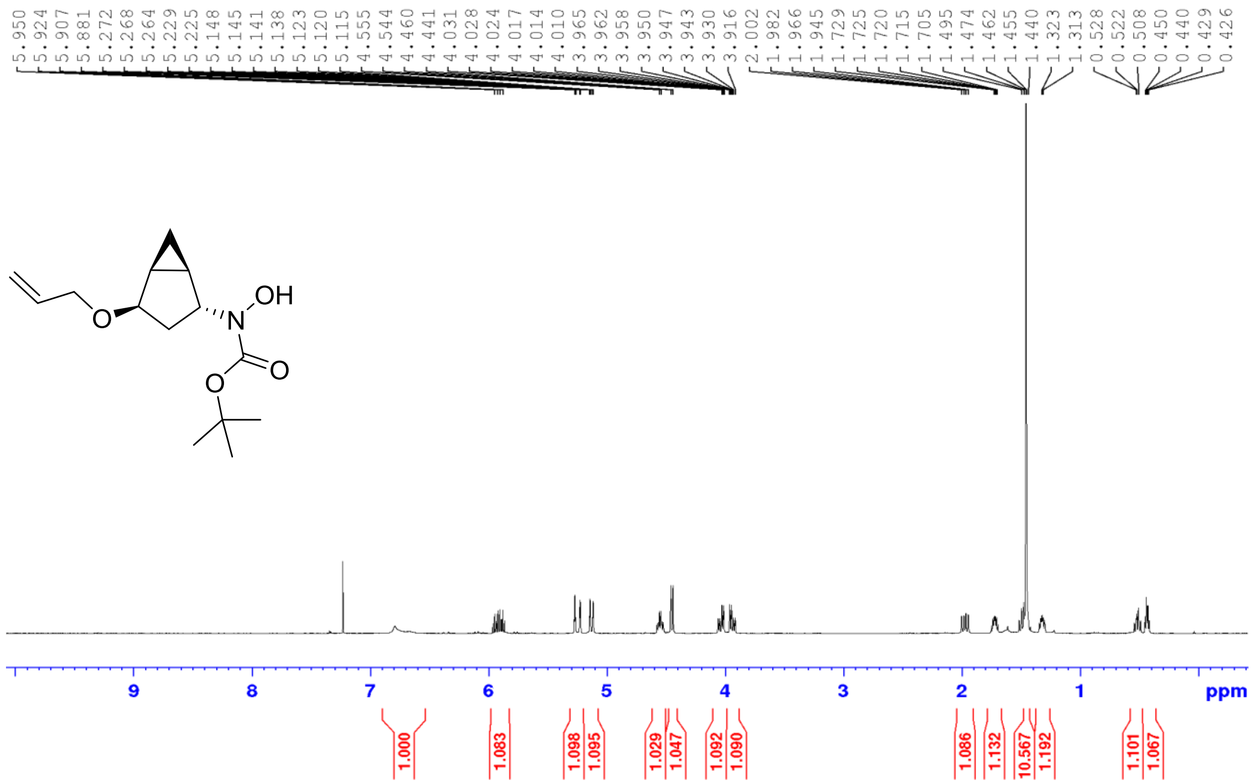

400 MHz ^{1}H NMR spectrum of **26g** in $CDCl_3$

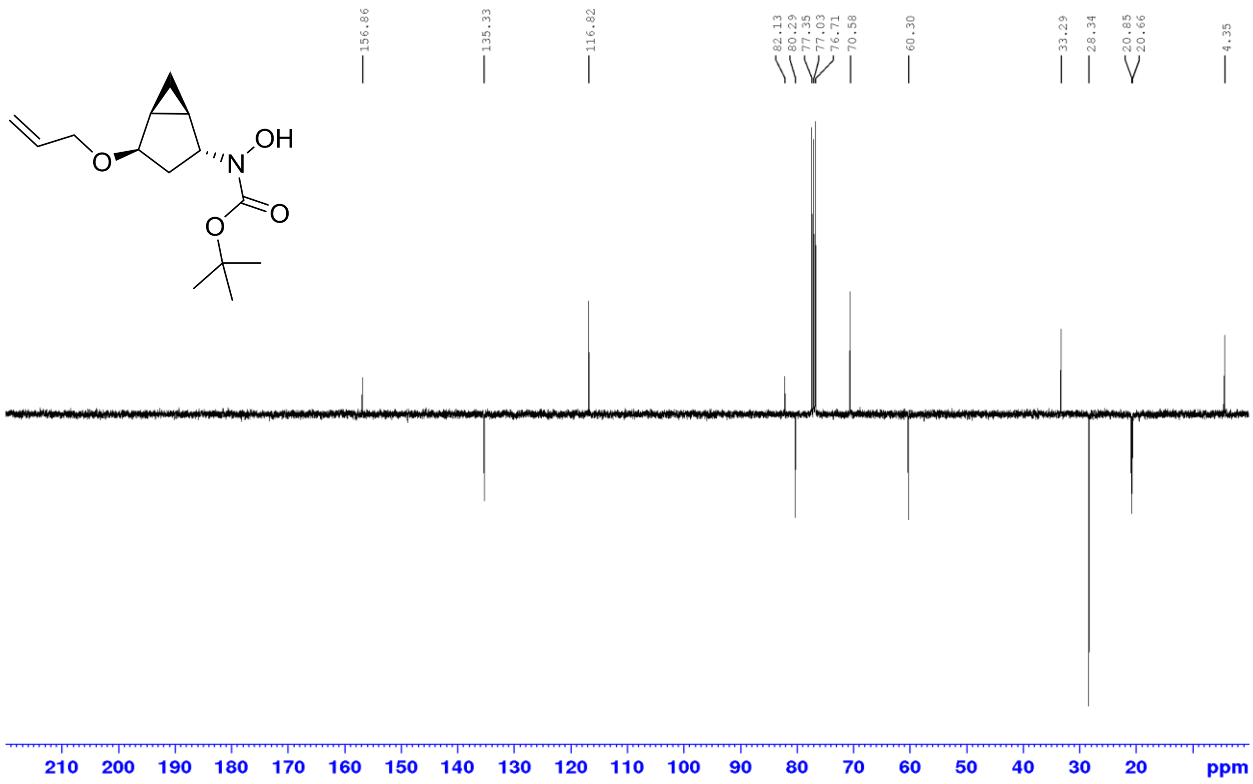

100 MHz ^{13}C NMR spectrum of **26g** in $CDCl_3$

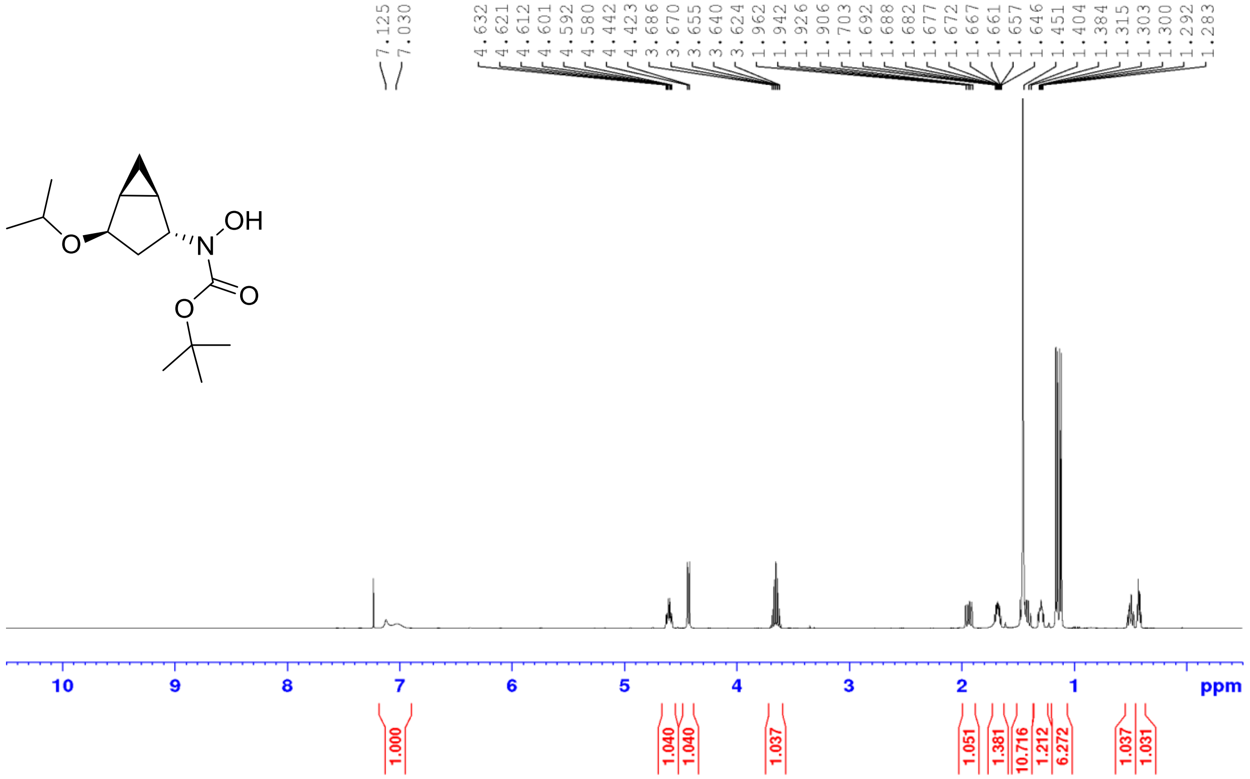

400 MHz ^{1}H NMR spectrum of **26h** in $CDCl_3$

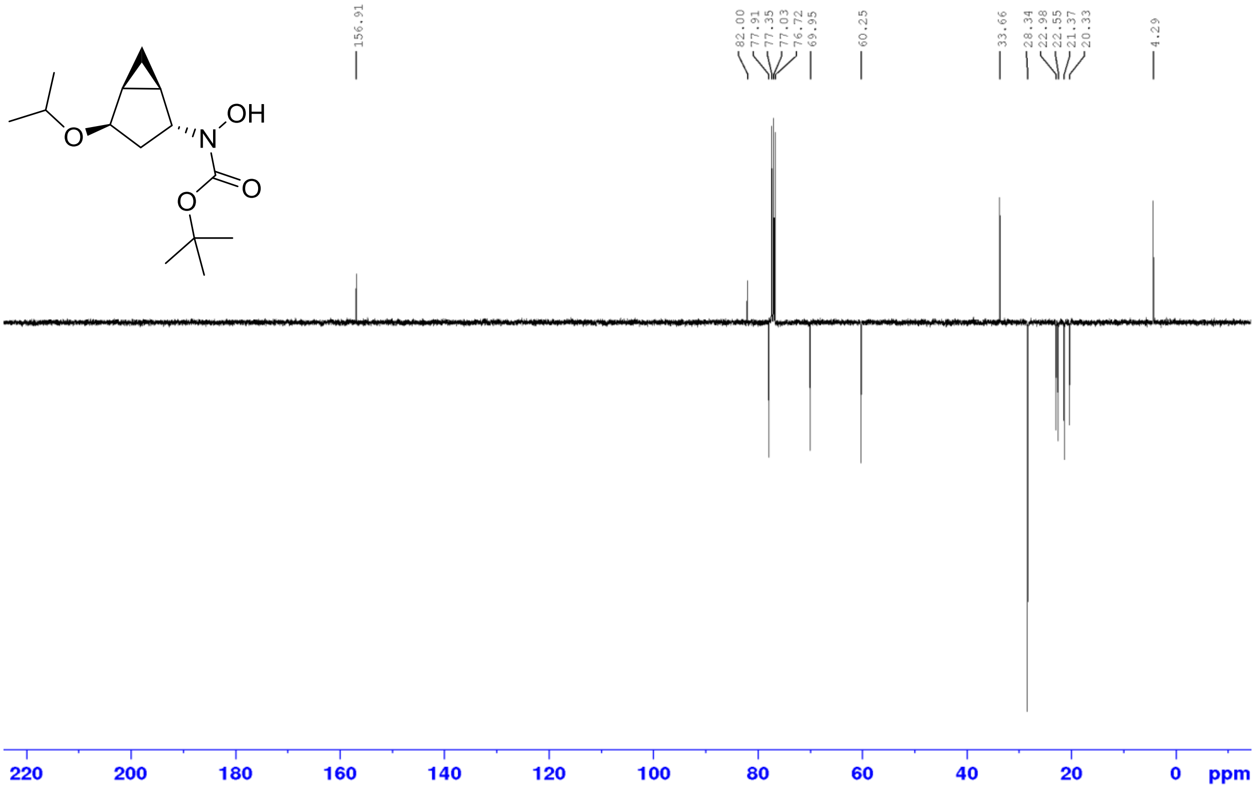

100 MHz ^{13}C NMR spectrum of **26h** in $CDCl_3$

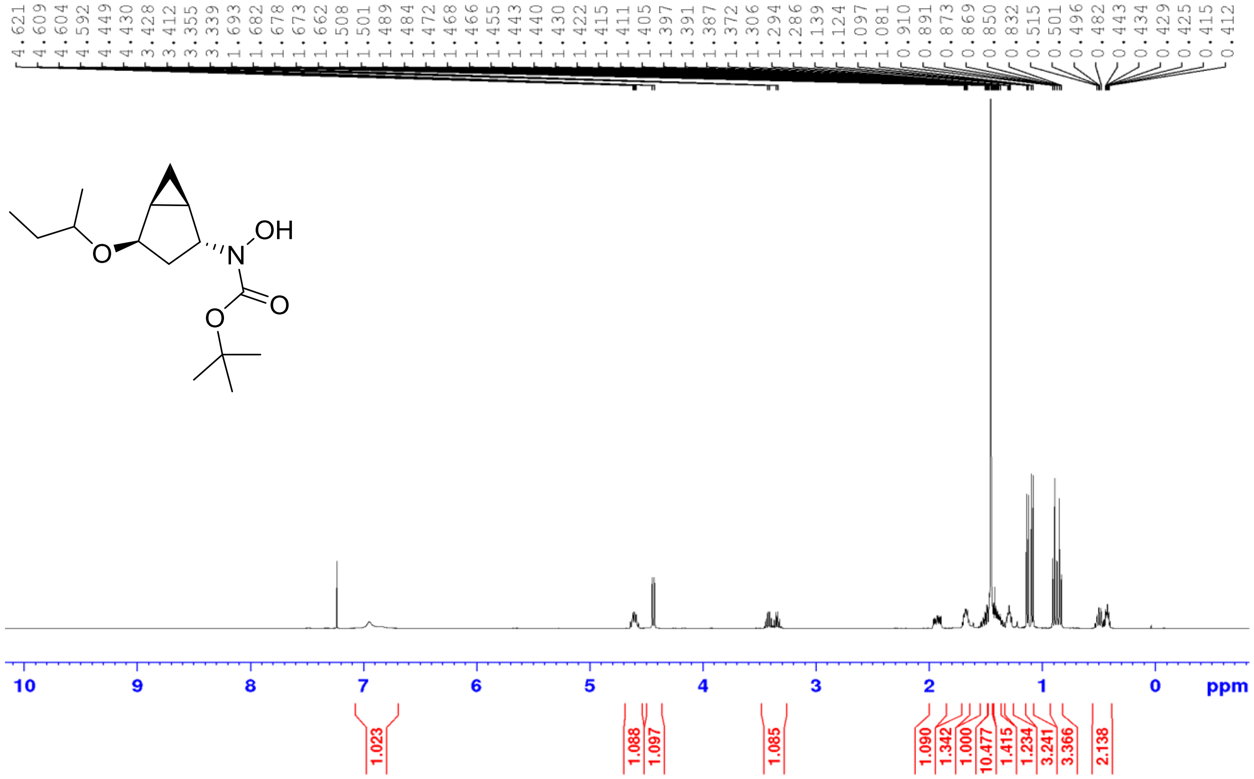

400 MHz ^{1}H NMR spectrum of **26i** in $CDCl_3$

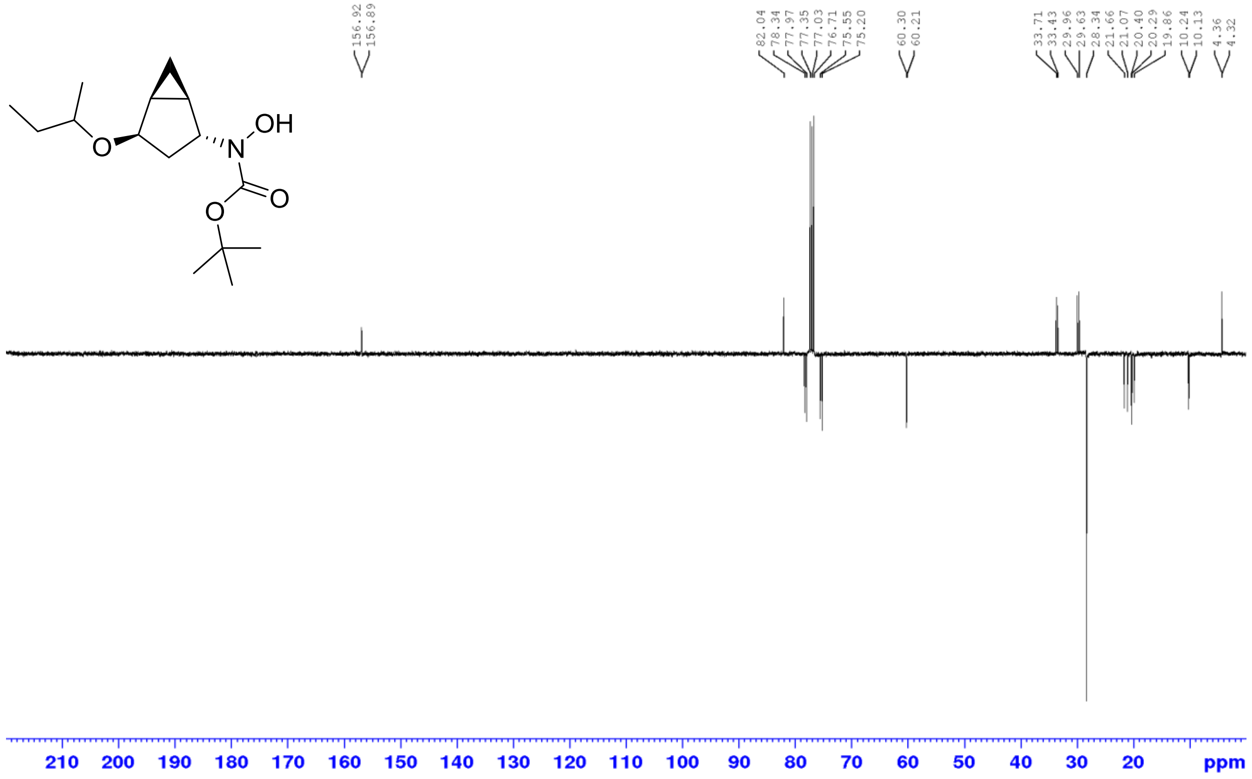

100 MHz ^{13}C NMR spectrum of **26i** in $CDCl_3$

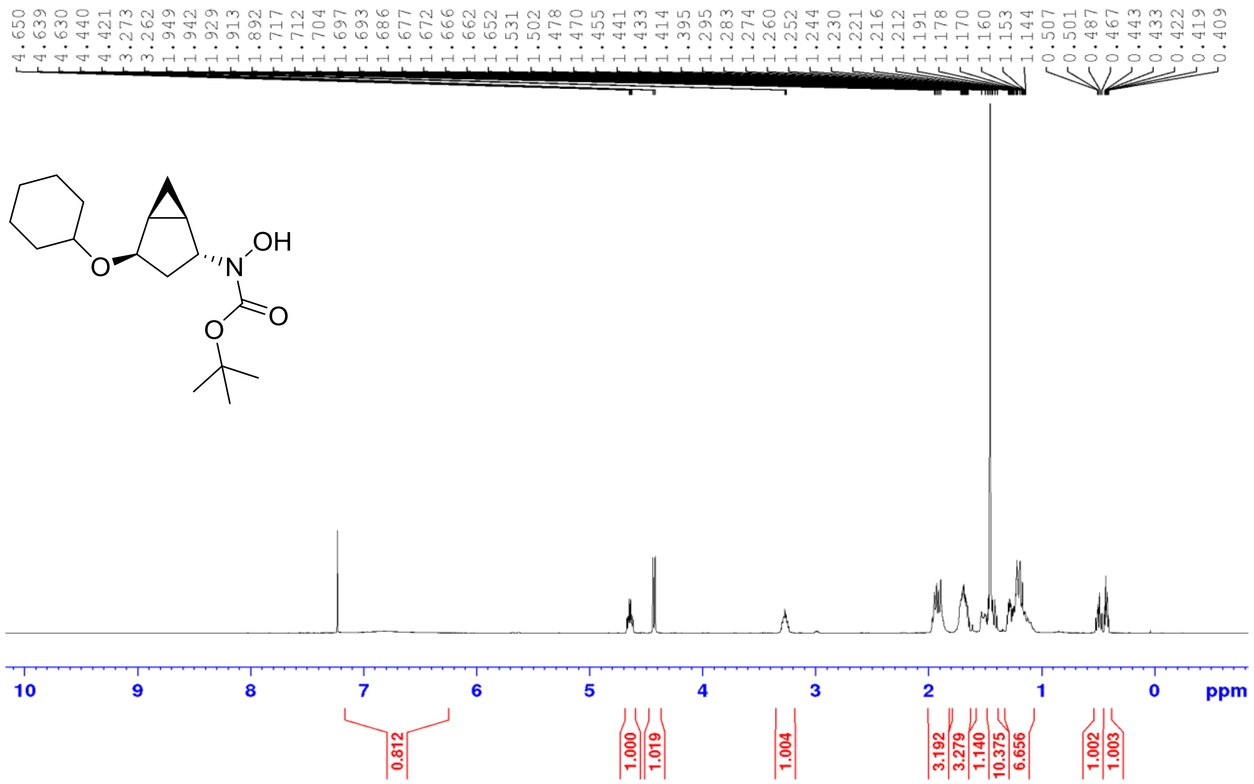

400 MHz ^{1}H NMR spectrum of **26j** in $CDCl_3$

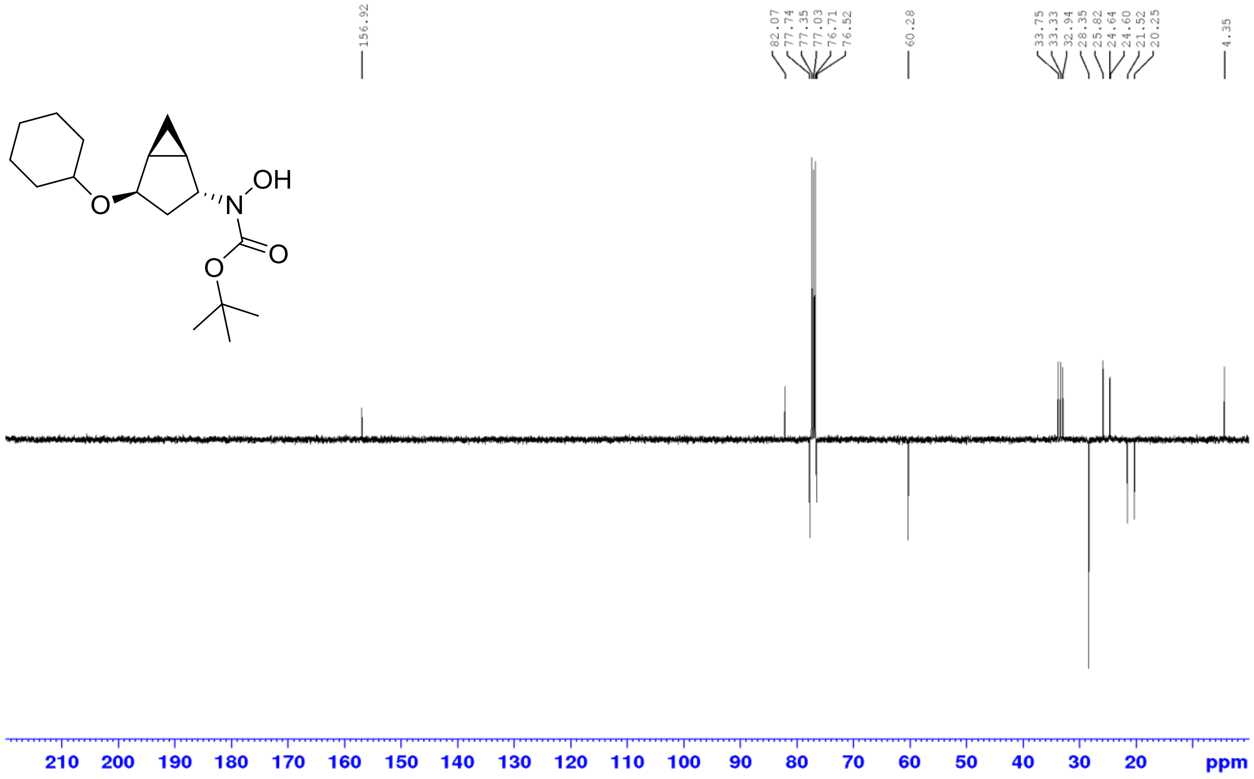

100 MHz ^{13}C NMR spectrum of **26j** in $CDCl_3$

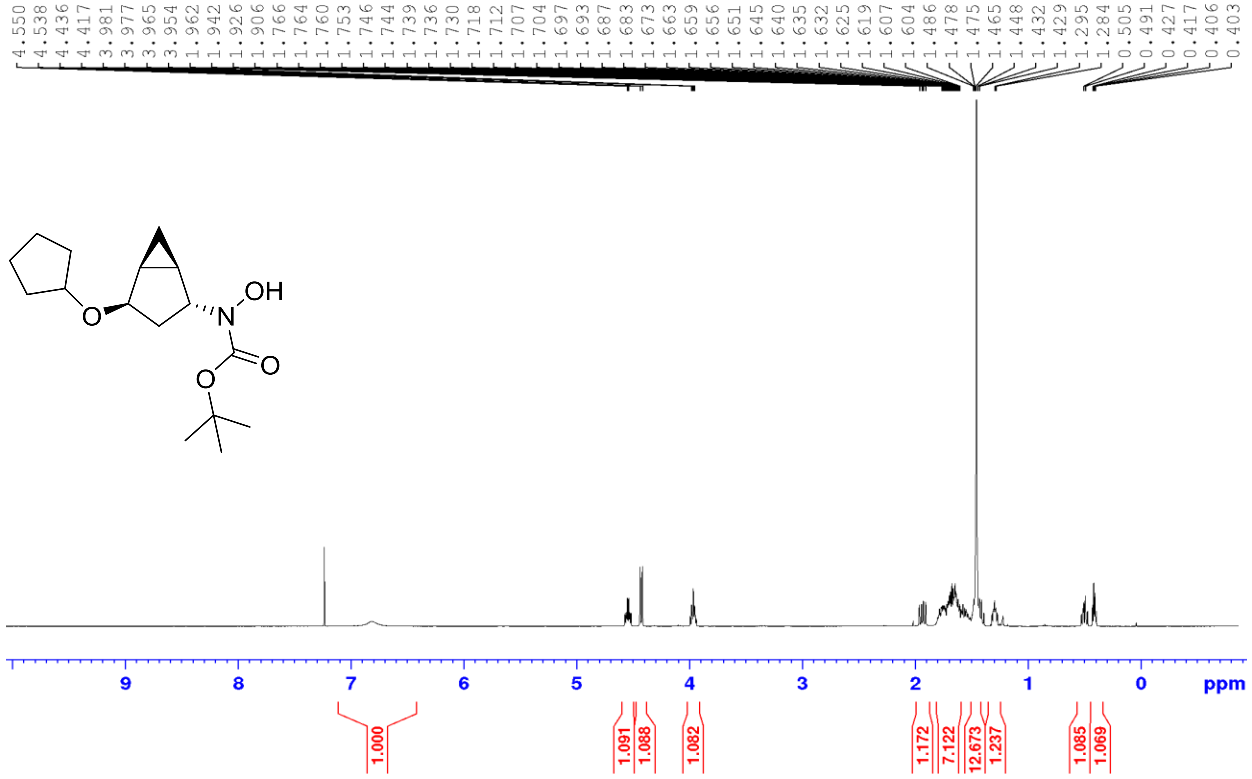

400 MHz ^{1}H NMR spectrum of **26k** in $CDCl_3$

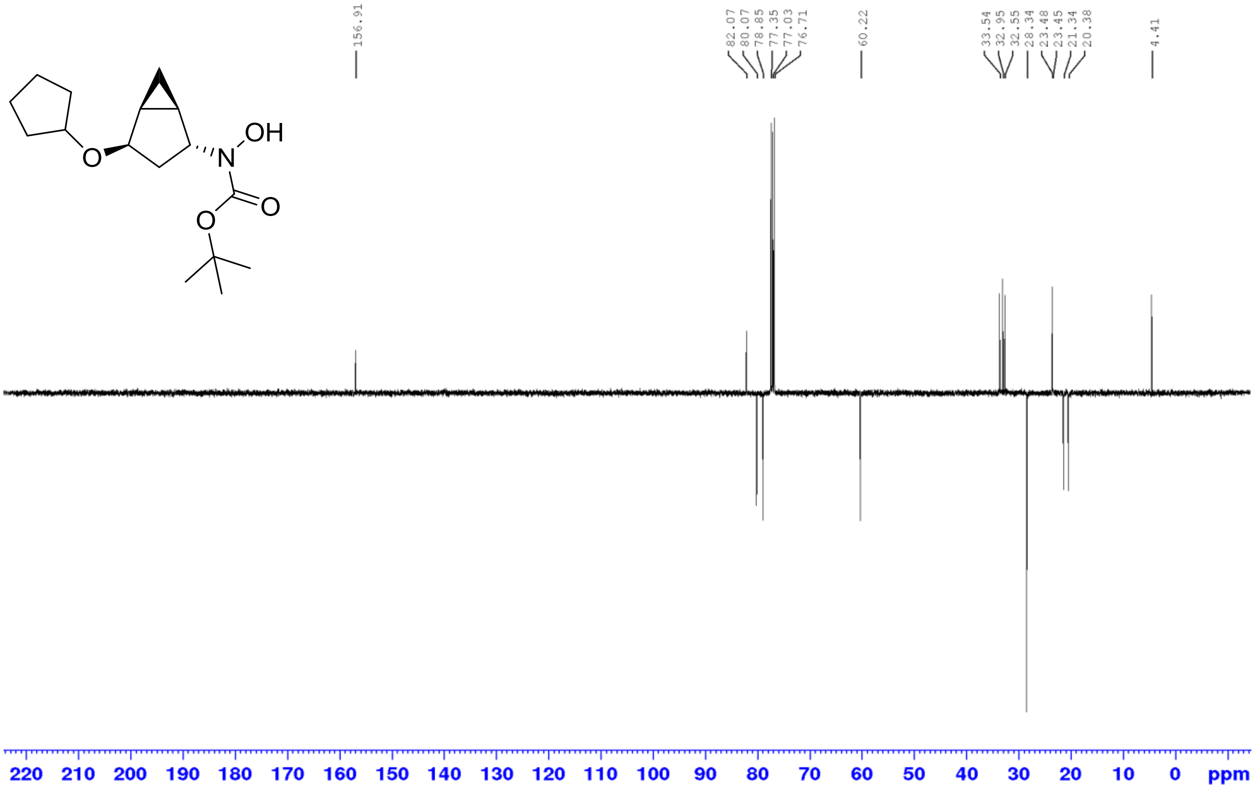

100 MHz ^{13}C NMR spectrum of **26k** in $CDCl_3$

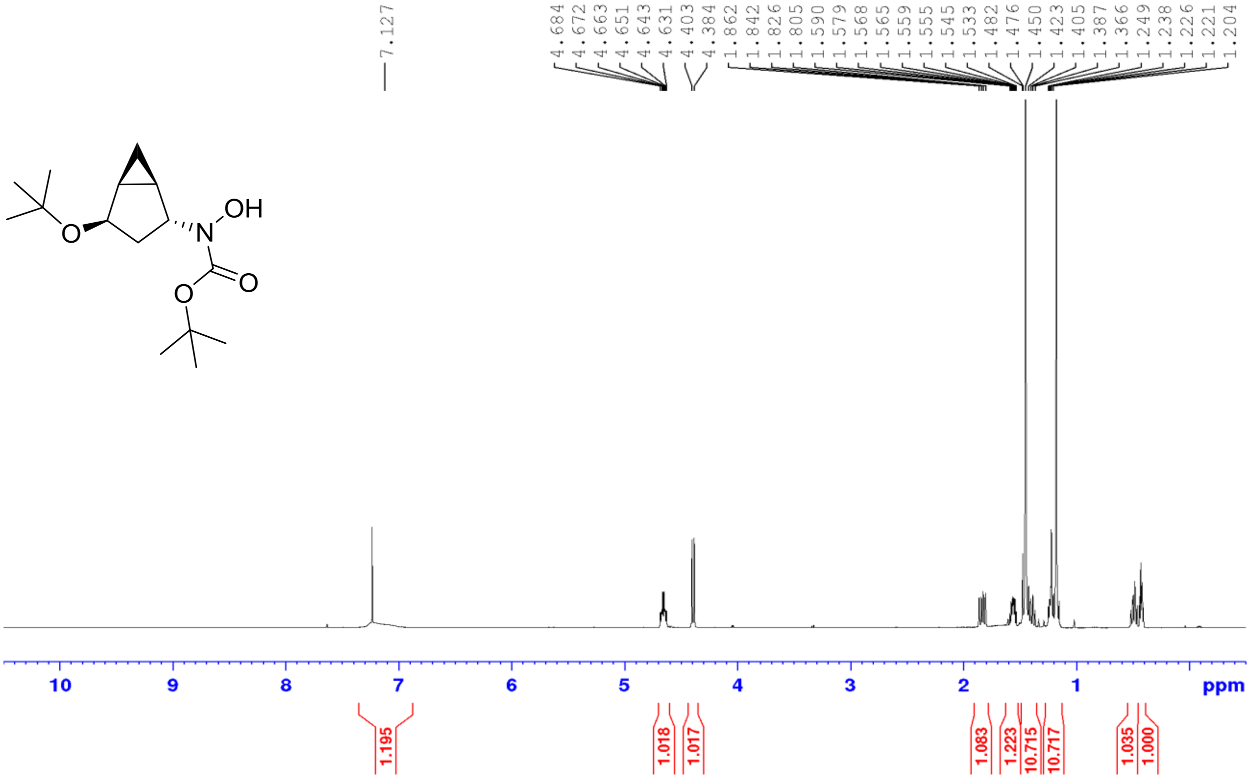

400 MHz ^{1}H NMR spectrum of **26l** in $CDCl_3$

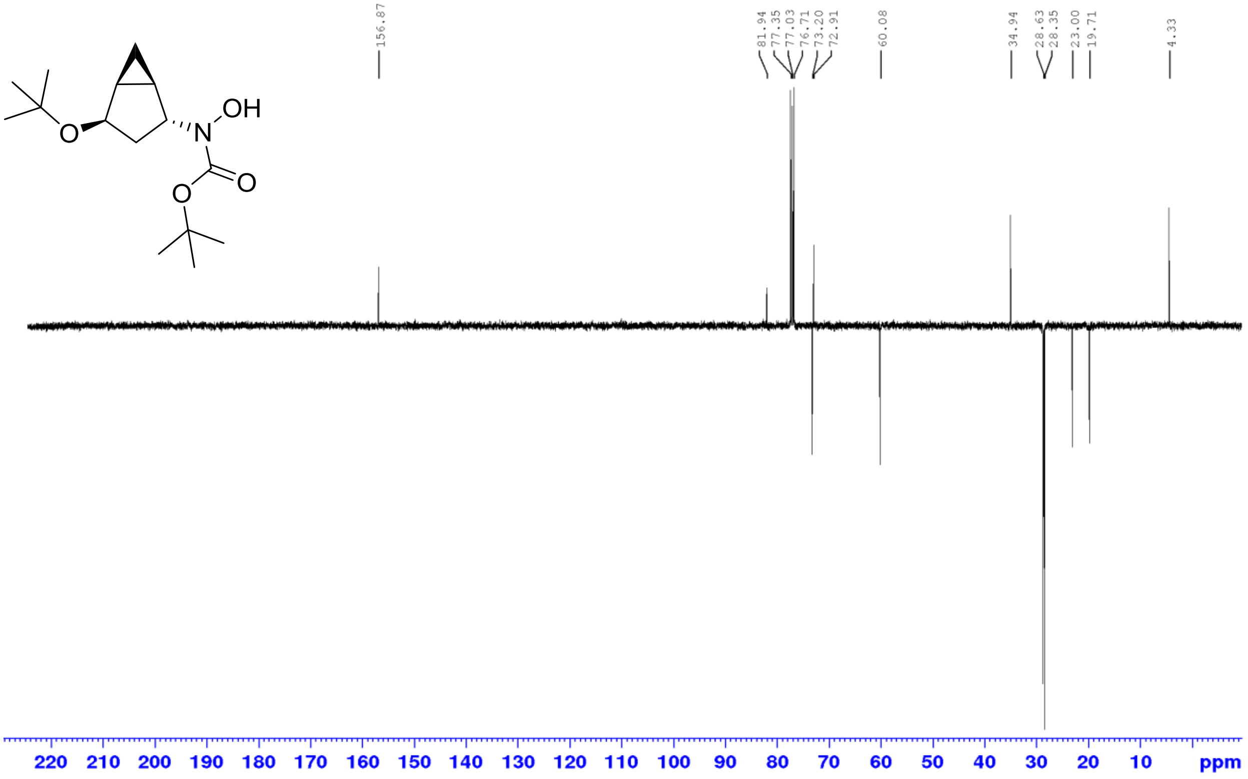

100 MHz ^{13}C NMR spectrum of **26l** in $CDCl_3$
